# Supplementary material for: Flexibility in Mannan-Binding Lectin-Associated Serine Proteases-1 and -2 Provides Insight on Lectin Pathway Activation
Source: Structure. 2017 Feb 7;25(2):364–75. doi: 10.1016/j.str.2016.12.014 (PMC5300068; doi:10.1016/j.str.2016.12.014)
Supplement: Document S1. Supplemental Experimental Procedures, Figures S1 and S2, Table S1 [file mmc1.pdf]

Structure, Volume *25*

Supplemental Information

Flexibility in Mannan-Binding Lectin-Associated Serine Proteases-1 and -2 Provides Insight on Lectin Pathway Activation

Ruodan Nan (南若丹), Christopher M. Furze, David W. Wright, Jayesh Gor, Russell Wallis, and Stephen J. Perkins

SUPPLEMENTARY INFORMATION

FLEXIBILITY IN MANNAN-BINDING LECTIN-ASSOCIATED SERINE PROTEASES-1 AND -2 PROVIDES NOVEL INSIGHT ON LECTIN PATHWAY ACTIVATION

Ruodan Nan (南若丹)[1], Christopher M. Furze[2], David W. Wright[1], Jayesh Gor[1], Russell Wallis[2], and Stephen J. Perkins[1]

From the [1]Department of Structural and Molecular Biology, Division of Biosciences, Darwin Building, University College London, Gower Street, London WC1E 6BT, U.K.; [2]Departments of Infection, Immunity and Inflammation and Molecular Cell Biology, Maurice Shock Medical Sciences Building, University of Leicester, University Road, Leicester, LE1 9HN, U.K.

***Contact**: Prof S. J. Perkins, Department of Structural and Molecular Biology, Darwin Building, University College London, Gower Street, London, WC1E 6BT, U.K. Tel: 020-7679-7048, FAX: 020-7679-7193; Email: s.perkins@ucl.ac.uk.

Table S1, related to Figure 8 and Figure S2. The mean values for the distances *D1*, *D2* and *D3* in the six MASP proteins, and the angles *θ1*, *θ2* and *θ3* for the starting structures and the best-fit models.

	Starting structures *				Ten best-fit models *			
	Distance (nm)		Angle (°)		Distance (nm)		Angle (°)	
	Mean *D1* and *D2*	*D3*	*θ3*	*θ1* and *θ2*	Mean *D1* and *D2*	*D3*	*θ3*	*θ1* and *θ2*
MASP-1 3D	5.0	10.0	171	4.5	5.1 ± 0.2	9.5 ± 0.5	142 ± 8	19 ± 4
MASP-2 3D	4.9	9.8	168	6.0	4.9 ± 0.2	9.5 ± 0.3	156 ± 9	12 ± 4
Zymogen MASP-1	14.3	28.6	172	3.9	12.0 ± 0.6	20.3 ± 0.9	117 ± 7	32 ± 3
Activated MASP-1	13.3	26.5	168	5.9	11.9 ± 1.2	22.4 ± 1.1	142 ± 13	19 ± 7
Zymogen MASP-2	12.4	24.7	166	7.1	10.8 ± 2.5	16.2 ± 2.1	100 ± 29	40 ± 19
Activated MASP-2	12.3	24.4	166	7.0	11.2 ± 1.7	20.1 ± 1.3	127 ± 10	26 ± 7

* The distances *D1*, *D2* and *D3* and the angles *θ1*, *θ2* and *θ3* for the starting structures and the best-fit models are defined in Figure S2. The values for full-length MASP-1 and MASP-2 are from Search 1.

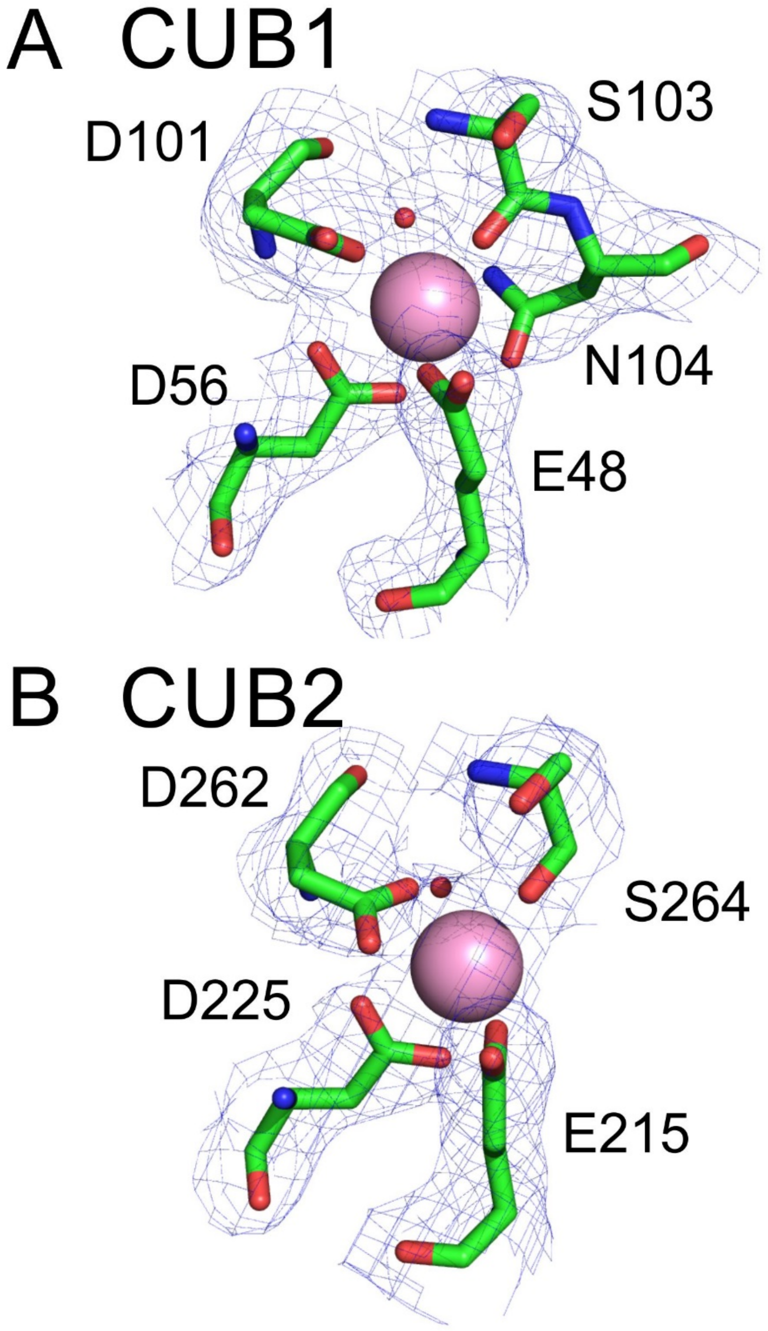

Figure S1, related to Figure 3B. Structure of the calcium binding sites in the CUB1 (A) and CUB2 (B) domains of MASP-2 from the structure PDB code 5CIS. The Ca^{2+} atom is shown as a pink sphere; a single water molecule is shown as a small red sphere. The electron density map is shown by the blue mesh ($\sigma = 1$).

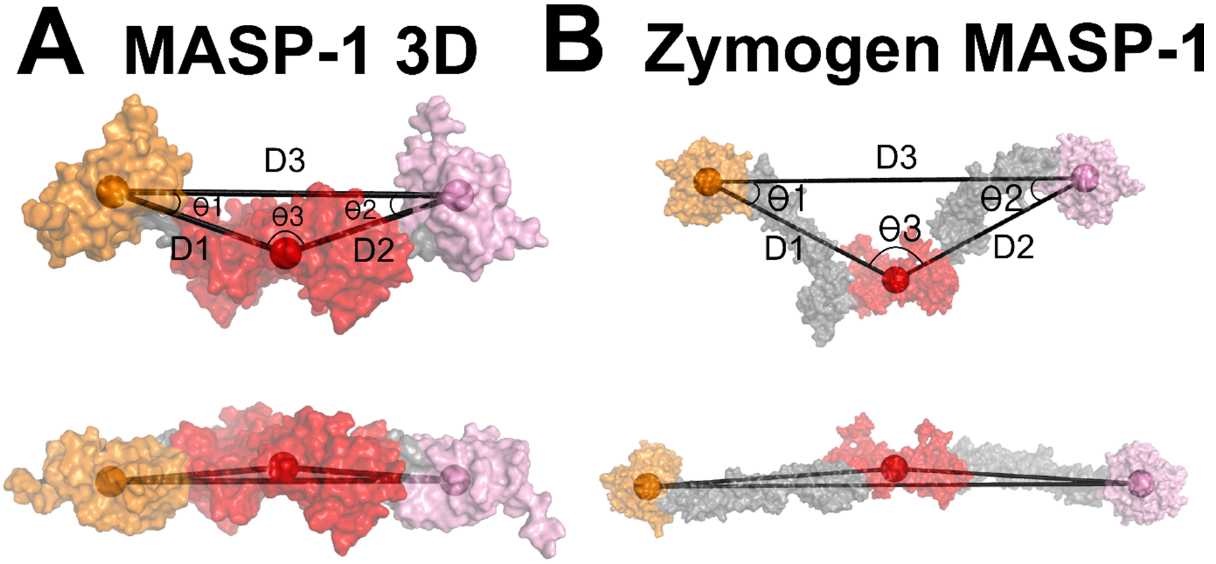

Figure S2, related to Figure 8 and Table S1. Comparison of the starting structure and best-fit models for MASP 3D and MASP. The distances D1-D3 and angles θ1-θ3 between the central and end regions of the MASP models defined their overall shapes (Table S1). *A*, The side-on view of the best-fit structure of MASP-1 3D (upper) is representative of MASP-1 3D and MASP-2 3D. This is compared in the same view with its crystal structure (lower). Its centre of mass is defined as the centre of the four CUB1-EGF domains (red). Two more centres of mass are defined as the CUB2 domain of monomer 1 (Arm 1; orange), and the CUB2 domain of monomer 2 (Arm 2; pink). *B*, The side-on view of the best-fit zymogen MASP-1 structure (upper) is representative of full-length MASP-1 and MASP-2. This is compared in the same view with its starting homology model used to initiate the scattering fits (lower). Its centre of mass is the centre of the four CUB1-EGF domains (red). Two more centres of mass are defined in the serine protease domains of monomer 1 (orange) and monomer 2 (pink).

EXPERIMENTAL PROCEDURES

Production, purification and compositions of the MASP proteins

In this study, all sequences were numbered according to the first residue in the mature protein, and not from the start of the signal peptide in accordance with previous structural analysis of MASPs. The rat zymogen MASP-1 was produced in Chinese hamster ovary cells by substituting the five residues immediately preceding the cleavage site for MASP-1 activation 425-KHISR-429 with DDDDK, where the latter is the recognition sequence for the serine protease enterokinase (Figure 1) (Chen and Wallis, 2001, 2004). This substitution prevented MASP-1 auto-activation. The rat zymogen MASP-2 form was produced by mutating Ser613 at its catalytic active site to Ala613 (Figure 1). Both zymogen MASP-1 and MASP-2 were synthesised with N-terminal hexahistidine tags and purified by nickel affinity chromatography, followed by size-exclusion gel filtration to remove aggregates and minor contaminants (Chen and Wallis, 2001, 2004). MASP-1 was activated by incubating the zymogen MASP-1 with enterokinase at a ratio of 0.01% w/w at 37°C in 20 mM Tris, 140 mM NaCl, 2 mM $CaCl_2$, pH 7.4 in a water bath for 4 h. The reaction was stopped by adding 1% w/w soybean trypsin inhibitor. MASP-2 was activated by incubating the zymogen MASP-2 at 1 mg/ml mixed with bovine trypsin at 10 µg/ml in 20 mM Tris, 140 mM NaCl, 0.1 mM EDTA, pH 7.4, for 210 s at 37°C in a water bath. The reaction was stopped by adding 20 µg/ml soybean trypsin inhibitor to the mixture and placing the reaction mixture immediately on ice. All proteins were purified by gel filtration chromatography on a Superdex 200 16/60 column (GE Healthcare), prior to crystallisation or further analysis. SDS-PAGE was used to check the purity of all the samples.

Zymogen MASP-1 and MASP-2 migrated as single band at about 80 kDa by SDS-PAGE under both reducing and non-reducing conditions. On activation, MASP-1 and MASP-2 showed a single band at 80 kDa in non-reducing conditions, and two bands at 30 kDa and 50 kDa in reducing conditions that correspond to the cleavage site just before the SP domain, which is disulphide linked but separate from the other five MASP domains. The MASP-1 3D and MASP-2 3D fragments showed two closely-positioned bands at 40 kDa, which were attributed to alternatively-glycosylated forms. Thus the major bands for the MASP monomers observed by SDS-PAGE confirm the purifications of disulphide-linked zymogen and activated MASP as previously described (Chen and Wallis, 2001; Wallis and Dodd, 2000). The absorbance coefficients (1%, 280 nm, 1 cm path length) for MASP-1 3D, zymogen MASP-1, activated MASP-1, MASP-2 3D, zymogen MASP-2 and activated MASP-2 were calculated to be 12.1, 14.5, 14.5, 12.3, 14.8 and 14.8 respectively. Molecular masses were calculated from the sequences to be 74.9 kDa for MASP-1 3D, 169.9 kDa for zymogen and activated MASP-1, 73.7 kDa for MASP-2 3D, and 162.7 kDa for zymogen and activated MASP-2, all in their dimeric forms. These masses assumed that one N-linked biantennary type oligosaccharide was present at each of the Asn40, Asn169 and Asn398 sites in zymogen and activated MASP-1, and the Asn94, Asn276 and Asn632 sites in zymogen and activated MASP-2 (Figure 1).

MASP crystallization, data collection and analysis

Proteins in 20 mM Tris pH 7.5 containing 20 mM NaCl and 2 mM $CaCl_2$ were crystallised using the sitting-drop vapour diffusion method by mixing equal volumes of protein (4 - 10 mg/mL) and crystallisation buffer at 20°C. The MASP-2 3D fragment was crystallised in three different conditions: 100 mM Tris pH 8.0 containing 40% 2-methyl-2,4-pentanediol and 80 mM ammonium acetate; 100 mM Tris pH 8.0 containing 40% 2-methyl-2,4-pentanediol; and 200 mM sodium citrate and 100 mM Tris pH 8.5 containing 80 mM ammonium acetate and 20% polyethylene glycol 20K. The MASP-1 3D fragment was crystallised in 100 mM imidazole/2-(*N*-morpholino)ethanesulphonic acid buffer at pH 6.5, containing 20% ethylene glycol and 10% polyethylene glycol 8K together with a mixture of 30 mM diethylene glycol, 30 mM triethylene glycol, 30 mM tetraethylene glycol and 30 mM pentaethylene glycol. Crystals were stored in liquid nitrogen and were maintained at 100 K during data collection. Diffraction data were collected at Leicester on a rotating copper anode home X-ray source and at the Diamond Light Source, and were processed with XDS (Kabsch, 1993) or Mosflm (Battye et al., 2011). Phases were determined by molecular replacement with Phaser (McCoy et al., 2007) using the crystal structures of human MASP-1 (PDB code: 3DEM) and rat MASP-2 (PDB code: 1NTO) as search models. Models were optimized by cycles of manual refinement with Coot (Emsley and Cowtan, 2004) and

refinement in Refmac5, part of the CCP4 software suite (Collaborative Computational Project, 1994), and in Phenix (Adams et al., 2010). The final refined crystal structures were deposited into the Protein Data Bank with accession codes 5CKN (MASP-2 3D in acetate), 5CIS (MASP-2 3D in 20% polyethylene glycol), and 5CKM (MASP-2 3D in citrate) and 5CKQ (MASP-1 3D), in that order. Calculations of buried surface areas at the dimer interface were made from PISA analyses (Krissinel and Henrick, 2007).

For MASP-1, the 12 contact residues (i.e. showing more than 50% buried accessible surface areas) at the dimer interface were Phe10, Met44, His115, Met117, Ala118, His139, Asn140, Tyr141, Ile142, Tyr146, Ser148 and Phe151. These 12 contact residues comprised 79% of the total buried accessible surface area. For the three MASP-2 structures, these 15-18 dimer interface contact residues were determined to be (Pro6), Phe8, Phe36, Arg39, Tyr41, Thr43, Glu112, Phe114, Ala116, Ala117, (Tyr136), His138, Asn139, Tyr140, Leu141, (Tyr145), Ser147 and Val150. These 15-18 dimer contact residues comprised 80-89% of the total buried accessible surface area. The majority of these contacts were hydrophobic in character. Two hydrophilic exceptions in MASP-2 included Arg39 that formed a hydrogen bond with a C=O of Asn139 across the dimer interface, and Glu110 that made a salt bridge with Arg10 within the same MASP molecule. All these contact residues were present in the human MASP-1 and MASP-2 sequences except for Phe36, Tyr136, Tyr140 and Val150 in rat MASP-2, which were replaced by the similar Tyr, His, Tyr and Ala residues in human MASP-2, respectively. This provides further evidence that a well-defined MASP dimer was physiologically important in both the human and rat proteins.

X-ray scattering data for MASP

X-ray scattering curves *I(Q)* were acquired in two beam sessions on the BioSAXS robot at Instrument BM29 at the European Synchrotron Radiation Facility, Grenoble, France (Pernot et al., 2013; Round et al., 2015). Data were recorded using a CMOS hybrid pixel Pilatus 1M detector with a resolution of 981 x 1043 pixels (pixel size of 172 µm × 172 µm). One session was operated with a ring energy of 6.0 GeV in 7/8 + 1 multibunch mode, and the other session was operated with a ring energy of 6.0 GeV in 24*8 + 1 filling mode. The sample-detector distance was 2.867 m, the X-ray wavelength was 0.09919 nm, and the diameter of the flow cell quartz capillary was 1.8 mm in both sessions. Potential radiation damage was eliminated by the continuous movement of the sample in the flow cell during beam exposure, the use of 10 time frames with an exposure time of 1 s per frame, and on-line checks for the absence of radiation damage. The scattering data were collected for MASP-1 3D at 0.31-0.78 mg/ml, zymogen MASP-1 at 0.25-1.26 mg/ml, and activated MASP-1 at 0.26-0.43 mg/ml in Tris buffer with 2 mM Ca^{2+}. Data were collected for MASP-2 3D at 0.30-0.60 mg/ml, zymogen MASP-2 at 0.30-0.68 mg/ml, and activated MASP-2 at 0.29 mg/ml in Tris buffer with EDTA. The MASP-2 samples were also measured with Ca^{2+} present, when 4 mM $CaCl_2$ was added immediately before measurements for MASP-2 3D at 0.30-0.60 mg/ml, zymogen MASP-2 at 0.3-0.5 mg/ml, and activated MASP-2 at 0.29 mg/ml. Data reduction was performed using ISpyB software (De Maria Antolinos et al., 2015).

In a given solute-solvent contrast, the radius of gyration R_g corresponds to the mean square distance of scattering elements from their centre of gravity, and is a measure of structural elongation. Guinier analyses of the *I(Q)* curves at low scattering vectors Q (where $Q = 4\pi \sin \theta/\lambda$; 2θ is the scattering angle and λ is the wavelength) give the R_g value and the forward scattering at zero angle $I(0)$ from the expression (Glatter and Kratky, 1982):

$$\ln I(Q) = \ln I(0) - R_g^2 Q^2/3$$

This expression is valid in a $Q.R_g$ range up to 1.5. Linear R_g plots were obtained in Q ranges of 0.23-0.38 nm^{-1} for both MASP 3Ds and 0.08-0.175 nm^{-1} for the full-length MASPs. If the structure is elongated, For elongated structures, the cross-sectional radius of gyration R_{xs} and cross-sectional intensity at zero angle $[I(Q)Q]_{Q\to0}$ is obtained from analyses at larger Q ranges:

$$\ln [I(Q)Q] = [I(Q)Q]_{Q\to0} - R_{xs}^2 Q^2/2$$

For MASP 3D, the linear R_{xs} region corresponded to a Q range of 0.45-0.75 nm^{-1}. For the full-length zymogen and activated MASP, the four $I(Q).Q$ vs Q^2 plots showed linear R_{xs} regions in the Q range of

0.60-0.86 nm^{-1}. The Guinier analyses were performed using SCT software (Miller et al., 2012; Wright and Perkins, 2015).

Indirect transformation of the $I(Q)$ curve measured in reciprocal space into real space gives the distance distribution function $P(r)$ and was carried out using the program GNOM (Semenyuk and Svergun, 1991):

$$P(r) = \frac{1}{2\pi^2} \int_o^\infty I(Q)\ Qr\ \sin(Qr)\ dQ$$

$P(r)$ corresponds to the distribution of distances r between volume elements, and gives an alternative calculation of the R_G and $I(0)$ values based on the full scattering curve $I(Q)$ following an assumption of the maximum dimension (D_{max}). This provided structural information in real space. It also gives the most frequently occurring distance M and the maximum dimension of the macromolecule L. For the MASP proteins, the X-ray curves utilised up to 497 data points in the Q range from 0.06 nm^{-1} to 2.20 nm^{-1} for MASP-1 3D and MASP-2 3D, from 0.14 nm^{-1} to 2.20 nm^{-1} for zymogen and activated MASP-1 and MASP-2. Other details are described elsewhere (Fernando et al., 2007; Nan et al., 2008; Okemefuna et al., 2008).

Sedimentation velocity data for MASP

Analytical ultracentrifugation data were obtained on Beckman XL-I instruments equipped with AnTi50 rotors and using two-sector cells with column heights of 12 mm at a rotor speed of 50,000 rpm. Sedimentation velocity experiments were performed at 20 °C for MASP-1 3D at 0.71 mg/ml, zymogen MASP-1 at 0.28 mg/ml, activated MASP-1 at 0.34 mg/ml (all three in 20 mM Tris, 140 mM NaCl, 2 mM $CaCl_2$, pH 7.4), and for MASP-2 3D at 0.32 mg/ml, zymogen MASP-2 at 0.14 mg/ml, and activated MASP-2 at 0.14 mg/ml (all three in a Tris buffer with EDTA, namely 20 mM Tris, 140 mM NaCl, 0.1 mM EDTA, pH 7.4). Sedimentation was monitored using absorbance optics at 280 nm. The sedimentation coefficient distribution $c(s)$ analyses were performed by fitting up to 60 absorbance scans directly to the Lamm equation using SEDFIT software version 14.6e (Schuck, 1998, 2000). In the $c(s)$ analyses, the meniscus, the bottom of the cell, the baseline, and the average frictional ratio f/f_0 were floated until the overall root mean deviation and the fits between the observed and calculated sedimentation boundaries were satisfactory. Here, f is the frictional coefficient of the macromolecule and f_0 is the frictional coefficient of the sphere with the same hydrated volume as the macromolecule. The starting f/f_0 values were 1.50 for MASP-1 3D and MASP-2 3D, and 1.85 for zymogen and activated MASP-1 and MASP-2. The partial specific volume v values were as follows: MASP-1 3D, 0.709 ml/g; zymogen MASP-1, 0.721 ml/g; activated MASP-1, 0.721 ml/g; MASP-2 3D, 0.715 ml/g; zymogen MASP-2, 0.725 ml/g; and activated MASP-2, 0.725 ml/g (Perkins, 1986). The buffer densities were measured using an Anton Paar DMA 5000 density meter, and compared with the theoretical values calculated by SEDNTERP (Laue et al., 1992). This resulted in a density of 1.00576 g/ml for Tris-Ca buffer at 20°C, compared to a theoretical value of 1.00576 g/ml, and one of 1.00559 g/ml for Tris-EDTA at 20°C.

Generation of starting structural models for MASP

Atomistic scattering modelling fits for each of the six proteins was performed in order to define their solution structures. For each protein, a starting model was constructed as follows, after which each model was subjected to two sets of conformational randomisation to generate broad ranges of trial structures for assessment. For each protein, the first search (Search 1) was initiated directly from the starting model detailed below. The second search (Search 2), was performed as a control of reproducibility and was seeded with the best fit scattering models from Search 1:

(i,ii) Rat MASP-1 3D and rat MASP-2 3D: For these initial $s_{20,w}$ calculations and scattering curve fits, the starting structures were the crystal structures for rat MASP-1 3D and rat MASP-2 3D bound with Ca^{2+} from this study (Figure 2).

(iii) Rat zymogen MASP-1 dimer: For the initial fits, the starting structure was based on three experimental structures: (a) the crystal structure for rat MASP-1 3D with Ca^{2+} (Figure 2*A*), (b) the

crystal structure for human mannose-binding lectin (MBL)/ficolin-associated protein-1 (MAP-1) which is the result of a differential splicing of MASP-1 gene and includes the CUB1-EGF-CUB2-SCR1 fragment of MASP-1 (PDB code: 4AQB) (Skjoedt et al., 2012a), and (c) the crystal structure of the SCR1-SCR2-SP fragment of human MASP-1 proenzyme (PDB code: 3GOV) (Dobo et al., 2009). Using MODELLER v9.11 (Sali and Blundell, 1993), a homology model for the rat MASP-1 CUB2-SCR1 fragment was constructed from the CUB2-SCR1 fragment of human MAP-1 (PDB code: 4AQB) as template. The homology model for the rat zymogen MASP-1 SCR1-SCR2-SP fragment was constructed from this fragment in the human MASP-1 crystal structure (PDB code: 3GOV). The homology model for the full-length rat zymogen MASP-1 monomer was formed using multi-templates to make the full monomer from structures for each of the three CUB1-EGF-CUB2, CUB2-SCR2 and SCR1-SCR2-SP fragments. The resulting monomer structure was superimposed onto the CUB1-EGF dimer in the crystal structure of MASP-1 3D dimer in order to form the full-length dimer (Figure 2*A*).

(iv) Activated MASP-1 dimer: The starting dimer structure was constructed using the same strategy for the zymogen, but replacing the template SCR1-SCR2-SP structure with the crystal structure of the SCR1-SCR2-SP fragment in catalytically active human MASP-1 (PDB code: 4DJZ) (Heja et al., 2012).

(v) Zymogen MASP-2 dimer: The starting structure was constructed in the same way as for MASP-1. The full-length rat zymogen MASP-2 structure was formed using the crystal structure for the rat MASP-2 3D dimer with Ca^{2+} (Figure 2*B*), combined with two rat homology models for the CUB2-SCR1 fragment based on the crystal structure of human MAP-1 (PDB code: 4AQB) and the SCR1-SCR2-SP fragment based on the crystal structure for human MASP-2 in its proenzyme form (PDB code: 1ZJK) (Gal et al., 2005).

(vi) Activated MASP-2 dimer: The starting structure for the homology model was based on the crystal structure of rat MASP-2 3D (Figure 2*B*). This was supplemented with three homology models constructed from the CUB2-SCR1 fragment in the crystal structure for human MAP-1 (PDB code: 4AQB), the SCR1-SCR2-SP fragment in the crystal structure for the human MASP-2 proenzyme (PDB code: 1ZJK) (Gal et al., 2005), and the crystal structure of the catalytically active human MASP-2 SCR2-SP fragment (PDB code: 3TVJ) (Heja et al., 2012).

Scattering curve calculation using SCT

Scattering curve simulations involved the generation of up to 30,000 conformationally-randomised domain arrangements of the six MASP proteins, followed by scattering curve calculations. The calculated curves were compared with the experimental X-ray scattering curves in order to identify the best fit solution structures (Perkins et al., 2009; Perkins et al., 2011; Perkins et al., 2016). For this, hydrogen atoms were added to all the starting MASP structures, each of which was parameterized using the CHARMM36 forcefield (Guvench et al., 2009; Hatcher et al., 2009; MacKerell et al., 1998; Mackerell et al., 2004; Raman et al., 2010) via the CHARMM-GUI website (http://www.charmm-gui.org/) (Jo et al., 2008; Jo et al., 2011) in order to generate parameters for the following structure simulations. Likely regions of linker flexibility between the six MASP domains and the His-tag in the starting structures were identified in bold (Figure 1). For the rat MASP-1 3D dimer, the flexible linkers were set as 162-RVE-164, and 277-HHHHHH-282. For rat zymogen and activated MASP-1, the flexible linkers were 1-HHHHHH-6, 173-RVE-175, 288-AGNE-291, 354-KIVD-357, and 424-LPV-426. For the rat MASP-2 3D dimer, the flexible linkers were 163-AL-164, and 279-HHHHHH-284. For rat zymogen and activated MASP-2, the flexible linkers were 1-HHHHHH-6, 173-AL-174, 287-TAQP-290, 353-SIID-356 and 421-KPV-423. The flexible linker conformations were randomly sampled using the dihedral angle Monte Carlo module of the SASSIE package (Curtis et al., 2012). The backbone φ and ψ angles of the peptide bonds within these linkers were varied independently of one another. The maximum rotation angle steps for both φ and ψ angles were 80°. Because the linkers and domains were considered together when creating each trial MASP structure, steric overlaps were handled better, in which sterically-poor models were discarded by SASSIE at their creation, and not later as in the classic SCT package (Miller et al., 2012). The Monte Carlo simulations generated 4,517 trial atomistic models for MASP-1 3D, 7,662 and 8,358 models for Search 1 and 2 respectively for zymogen MASP-1, 30,910 and 8,685 models for Search 1 and Search 2 for activated MASP-1, 1,982 models for MASP-2 3D, 7,662 and 8,685 models for Search 1 and Search 2 for zymogen MASP-2, and 6,173 and 8,685 models from Search 1 and Search 2 for activated MASP-2.

Each set of models generated by the Monte Carlo simulations was submitted to the SCT software suite to calculate scattering curves for comparison to the experimental X-ray scattering data (Wright and Perkins, 2015). The models were coarse-grained into small sphere models using a grid with cube-side length of 0.530 nm and a cut-off of four atoms. These parameters were optimized by SCT to produce unhydrated protein models with the correct volume calculated from the sequence. For X-ray scattering curve calculations, a hydration sphere shell corresponding to 0.3 g of water per gram of glycoprotein was added to each unhydrated model (Perkins, 1986, 2001; Wright and Perkins, 2015). Scattering curves *I(Q)* were calculated from the hydrated sphere models using the Debye equation adapted to spheres (Wright and Perkins, 2015). The calculated scattering curves from the sphere models were then compared to the experimental scattering data using a goodness of fit *R*-factor in a *Q* range extending to 2.2 nm^{-1}. The Guinier R_g and R_{xs} values were calculated from the *I(Q)* curves from the sphere models using the same *Q* ranges used to determine the experimental R_g and R_{xs} values. In each fit analysis, the best ten models were selected using three filters, namely the R_g, R_{xs}, and *R*-factor values.

To complete each search fit, one N-linked biantennary type oligosaccharide was added to each of Asn-40, Asn-169 and Asn-398 for each best-fit model of zymogen and activated MASP-1. The core oligosaccharide structure was taken from the crystal structure of the Fc fragment of human IgG1 to which additional terminal residues were added (Boehm et al., 1999). For each best-fit model of zymogen and activated MASP-2, oligosaccharide was added to each of Asn-94, Asn-276 and Asn-632. These best-fit models with carbohydrate added were resubmitted to SCT to calculate the final R_G, R_{XS} and *R*-factor values.

Utility of atomistic scattering modelling

Atomistic scattering modelling offers unique views of the multidomain complement protein structures in solution, which reinforces the interpretation of crystal structures and provides important new insights (Rodriguez et al., 2015). This method has a good track record with 77 structure determinations (Wright and Perkins, 2015), as exemplified by four examples: (i) Our scattering modelling of the SCR-6/8 domains of complement factor H predated its crystal structure and both methods revealed the same inter-SCR domain arrangement (Fernando et al., 2007; Prosser et al., 2007); (ii) The J-shaped scattering structure of factor H SCR-1/5 was used to solve the crystal structure of its complex with C3b (Okemefuna et al., 2008; Wu et al., 2009); (iii) The J-shaped structure of the five-domain SCR-1/5 rat CrrY protein was confirmed by its subsequent crystal structure (Aslam et al., 2003; Roversi et al., 2011a); (iv) The scattering modelling of a compact five-domain structure for complement factor I was confirmed by its crystal structure 13 years later (Chamberlain et al., 1998; Roversi et al., 2011b).

For the MASP proteins, crystal structures for four N-terminal CUB1-EGF-CUB2 and six C-terminal SCR1-SCR2-SP fragments were previously available, together with one more showing how the CUB2-SCR1 domains were connected (Dobo et al., 2009; Feinberg et al., 2003; Gal et al., 2005; Gingras et al., 2011; Gregory et al., 2003; Harmat et al., 2004; Heja et al., 2012; Kidmose et al., 2012; Skjoedt et al. 2012; Teillet et al., 2008). The similarity of the domain arrangements in these structures gave rise to the expectation that MASP only has one single conformation. Here, the utility of atomistic modelling was two-fold. First, in MASP 3D, atomistic modelling revealed that the EGF-CUB2 junction exhibited varied structures in solution, implying considerable flexibility. Second, in the full-length MASP proteins, atomistic modelling indicated flexibility at potentially all four linkers (EGF-CUB2, CUB2-SCR1, SCR1-SCR2 and SCR2-SP), although it was not possible to identify the degree of flexibility at each linker. This flexibility, together with the glycosylation of MASP-1 and MASP2, may explain why the full-length MASP proteins have not been crystallised so far.

The present atomistic modelling of MASP benefitted from a new methodology SASSIE to generate physically-realistic and conformationally-randomised domain arrangements based on molecular dynamics and Monte Carlo simulations (Curtis et al., 2012; Wright and Perkins, 2015; Perkins et al., 2016). Earlier crystallography and scattering analyses revealed that human and mouse IgG antibodies were not necessarily two-fold symmetric (Harris et al., 1998; Rayner et al., 2014, 2015;

Saphire et al., 2001). Thus two-fold symmetry was not assumed for the MASP dimer modelling. During each SASSIE model generation, typically 30,000 randomised linker conformations in MASP-1 and MASP-2 were generated by Monte Carlo variations of 20 and 19 θ and ψ angles. Our earlier methodology produced 10-fold less models. Such a Monte Carlo approach is rapid; 50,000 structures were sampled in less than an hour on a single CPU. Such a single-search approach is simple and does not require a range of assumptions to create molecular models (Kjaer et al., 2015). Because the MASP domains were simultaneously considered together in each trial MASP structure, sterically-poor models showing overlaps were discarded at their creation, rather than later. The availability of a large number of randomised models improved the quality of the resulting curve fits in a Q range out to 1.5 nm^{-1} with an R factor as low as 4.1% (Figure 7). The present MASP analyses indicate that atomistic modelling will continue to be useful in structure-function studies of multidomain proteins.

REFERENCES

Adams, P.D., Afonine, P.V., Bunkoczi, G., Chen, V.B., Davis, I.W., Echols, N., Headd, J.J., Hung, L.W., Kapral, G.J., Grosse-Kunstleve, R.W., *et al.* (2010). PHENIX: a comprehensive Python-based system for macromolecular structure solution. Acta Cryst. D*66*, 213-221.

Aslam, M., Guthridge, J.M., Hack, B.K., Quigg, R.J., Holers, V.M., and Perkins, S.J. (2003). The extended multidomain solution structures of the complement protein Crry and its chimeric conjugate Crry-Ig by scattering, analytical ultracentrifugation and constrained modelling: implications for function and therapy. J. Mol. Biol. *329*, 525-550.

Battye, T.G., Kontogiannis, L., Johnson, O., Powell, H.R., and Leslie, A.G. (2011). iMOSFLM: a new graphical interface for diffraction-image processing with MOSFLM. Acta Cryst. D*67*, 271-281.

Boehm, M.K., Woof, J.M., Kerr, M.A., and Perkins, S.J. (1999). The Fab and Fc fragments of IgA1 exhibit a different arrangement from that in IgG: a study by X-ray and neutron solution scattering and homology modelling. J. Mol. Biol. *286*, 1421-1447.

Chamberlain, D., Ullman, C.G., and Perkins, S.J. (1998). Possible arrangement of the five domains in human complement factor I as determined by a combination of X-ray and neutron scattering and homology modeling. Biochemistry *37*, 13918-13929.

Chen, C.B., and Wallis, R. (2001). Stoichiometry of complexes between mannose-binding protein and its associated serine proteases. Defining functional units for complement activation. J. Biol. Chem. *276*, 25894-25902.

Chen, C.B., and Wallis, R. (2004). Two mechanisms for mannose-binding protein modulation of the activity of its associated serine proteases. J. Biol. Chem. *279*, 26058-26065.

Collaborative Computational Project, N. (1994). The CCP4 Suite: Programs for Protein Crystallography. Acta. Cryst. *D50*, 760-763.

Curtis, J.E., Raghunandan, S., Nanda, H., and Krueger, S. (2012). SASSIE: A program to study intrinsically disordered biological molecules and macromolecular ensembles using experimental scattering restraints. Comput. Phys. Commun *183*, 382-389.

De Maria Antolinos, A., Pernot, P., Brennich, M.E., Kieffer, J., Bowler, M.W., Delageniere, S., Ohlsson, S., Malbet Monaco, S., Ashton, A., Franke, D., *et al.* (2015). ISPyB for BioSAXS, the gateway to user autonomy in solution scattering experiments. Acta Cryst. D*71*, 76-85.

Dobo, J., Harmat, V., Beinrohr, L., Sebestyen, E., Zavodszky, P., and Gal, P. (2009). MASP-1, a promiscuous complement protease: structure of its catalytic region reveals the basis of its broad specificity. J. Immunol. *183*, 1207-1214.

Emsley, P., and Cowtan, K. (2004). Coot: model-building tools for molecular graphics. Acta Cryst. D*60*, 2126-2132.

Feinberg, H., Uitdehaag, J.C., Davies, J.M., Wallis, R., Drickamer, K., and Weis, W.I. (2003). Crystal structure of the CUB1-EGF-CUB2 region of mannose-binding protein associated serine protease-2. EMBO J. *22*, 2348-2359.

Fernando, A.N., Furtado, P.B., Clark, S.J., Gilbert, H.E., Day, A.J., Sim, R.B., and Perkins, S.J. (2007). Associative and structural properties of the region of complement factor H encompassing the Tyr402His disease-related polymorphism and its interactions with heparin. J. Mol. Biol. *368*, 564-581.

Gal, P., Harmat, V., Kocsis, A., Bian, T., Barna, L., Ambrus, G., Vegh, B., Balczer, J., Sim, R.B., Naray-Szabo, G., and Zavodszky, P. (2005). A true autoactivating enzyme. Structural insight into mannose-binding lectin-associated serine protease-2 activations. J. Biol. Chem. *280*, 33435-33444.

Gingras, A.R., Girija, U.V., Keeble, A.H., Panchal, R., Mitchell, D.A., Moody, P.C., and Wallis, R. (2011). Structural basis of mannan-binding lectin recognition by its associated serine protease MASP-1: implications for complement activation. Structure *19*, 1635-1643.

Glatter, O., and Kratky, O. (1982). Small angle X-ray scattering (New York: Academic Press).

Gregory, L.A., Thielens, N.M., Arlaud, G.J., Fontecilla-Camps, J.C., and Gaboriaud, C. (2003). X-ray structure of the Ca2+-binding interaction domain of C1s. Insights into the assembly of the C1 complex of complement. J. Biol. Chem. *278*, 32157-32164.

Guvench, O., Hatcher, E.R., Venable, R.M., Pastor, R.W., and Mackerell, A.D. (2009). CHARMM Additive All-Atom Force Field for Glycosidic Linkages between Hexopyranoses. J. Chem. Theory Comput. *5*, 2353-2370.

Harmat, V., Gal, P., Kardos, J., Szilagyi, K., Ambrus, G., Vegh, B., Naray-Szabo, G., and Zavodszky, P. (2004). The structure of MBL-associated serine protease-2 reveals that identical substrate specificities of C1s and MASP-2 are realized through different sets of enzyme-substrate interactions. J. Mol. Biol. *342*, 1533-1546.

Harris, L.J., Larson, S.B., Skaletsky, E., and McPherson, A. (1998). Comparison of the conformations of two intact monoclonal antibodies with hinges. Immunol. Rev. *163*, 35-43.

Hatcher, E., Guvench, O., and Mackerell, A.D., Jr. (2009). CHARMM Additive All-Atom Force Field for Acyclic Polyalcohols, Acyclic Carbohydrates and Inositol. J. Chem. Theory Computat. *5*, 1315-1327.

Heja, D., Kocsis, A., Dobo, J., Szilagyi, K., Szasz, R., Zavodszky, P., Pal, G., and Gal, P. (2012). Revised mechanism of complement lectin-pathway activation revealing the role of serine protease MASP-1 as the exclusive activator of MASP-2. Proc. Natl. Acad. Sci. USA *109*, 10498-10503.

Jo, S., Kim, T., Iyer, V.G., and Im, W. (2008). CHARMM-GUI: a web-based graphical user interface for CHARMM. J. Computat. Chem. *29*, 1859-1865.

Jo, S., Song, K.C., Desaire, H., MacKerell, A.D., Jr., and Im, W. (2011). Glycan Reader: automated sugar identification and simulation preparation for carbohydrates and glycoproteins. J. Computat. Chem. *32*, 3135-3141.

Kabsch, W. (1993). Automatic processing of rotation diffraction data from crystals of initially unknown symmetry and cell constants. J. Appl. Cryst. *26*, 795-800.

Kidmose, R.T., Laursen, N.S., Dobo, J., Kjaer, T.R., Sirotkina, S., Yatime, L., Sottrup-Jensen, L., Thiel, S., Gal, P., and Andersen, G.R. (2012). Structural basis for activation of the complement system by component C4 cleavage. Proc. Natl. Acad. Sci. USA *109*, 15425-15430.

Kjaer, T.R., Le le, T.M., Pedersen, J.S., Sander, B., Golas, M.M., Jensenius, J.C., Andersen, G.R., and Thiel, S. (2015). Structural insights into the initiating complex of the lectin pathway of complement activation. Structure *23*, 342-351.

Krissinel, E., and Henrick, K. (2007). Inference of macromolecular assemblies from crystalline state. J. Mol. Biol. *372*, 774-797.

Laue, T.M., Bhairavi, D.S., Ridgeway, T.M., and Pelletier, S.L. (1992). In Analytical Ultracentrifugation in Biochemistry and Polymers Science, S.E. Harding, A.J. Rowe, and J.C. Horton, eds. (Royal Society of Chemistry), pp. 90-125.

MacKerell, A.D., Bashford, D., Bellott, M., Dunbrack, R.L., Evanseck, J.D., Field, M.J., Fischer, S., Gao, J., Guo, H., Ha, S., *et al.* (1998). All-atom empirical potential for molecular modeling and dynamics studies of proteins. J. Phys. Chem. B *102*, 3586-3616.

Mackerell, A.D., Jr., Feig, M., and Brooks, C.L., 3rd (2004). Extending the treatment of backbone energetics in protein force fields: limitations of gas-phase quantum mechanics in reproducing protein conformational distributions in molecular dynamics simulations. J. Comput. Chem. *25*, 1400-1415.

McCoy, A.J., Grosse-Kunstleve, R.W., Adams, P.D., Winn, M.D., Storoni, L.C., and Read, R.J. (2007). Phaser crystallographic software. J. Appl. Cryst. *40*, 658-674.

Miller, A., Phillips, A., Gor, J., Wallis, R., and Perkins, S.J. (2012). Near-planar solution structures of mannose-binding lectin oligomers provide insight on the activation of the lectin pathway of complement. J. Biol. Chem. *287*, 3930-3945.

Nan, R., Gor, J., Lengyel, I., and Perkins, S.J. (2008). Uncontrolled zinc- and copper-induced oligomerisation of the human complement regulator factor H and its possible implications for function and disease. J. Mol. Biol. *384*, 1341-1352.

Okemefuna, A.I., Gilbert, H.E., Griggs, K.M., Ormsby, R.J., Gordon, D.L., and Perkins, S.J. (2008). The regulatory SCR-1/5 and cell surface-binding SCR-16/20 fragments of factor H reveal partially folded-back solution structures and different self-associative properties. J. Mol. Biol. *375*, 80-101.

Perkins, S.J. (1986). Protein volumes and hydration effects. The calculations of partial specific volumes, neutron scattering matchpoints and 280-nm absorption coefficients for proteins and glycoproteins from amino acid sequences. Eur. J. Biochem. *157*, 169-180.

Perkins, S.J. (2001). X-ray and neutron scattering analyses of hydration shells: a molecular interpretation based on sequence predictions and modelling fits. Biophys. Chem *93*, 129-139.

Perkins, S.J., Okemefuna, A.I., Nan, R., Li, K., and Bonner, A. (2009). Constrained solution scattering modelling of human antibodies and complement proteins reveals novel biological insights. J. Roy. Soc. Interface *6*, S679-696.

Perkins, S.J., Nan, R., Li, K., Khan, S., and Abe, Y. (2011). Analytical ultracentrifugation combined with X-ray and neutron scattering: Experiment and modelling. Methods *54*, 181-199.

Perkins, S.J., Wright, D.W., Zhang, H., Brookes, E.H., Chen, J., Irving, T.C., Krueger, S., Barlow, D.J., Edler, K.J., Scott, D.J. *et al.* (2016) Atomistic modelling of scattering data in the Collaborative Computational Project for Small Angle Scattering (CCP-SAS). J. App. Crystallogr. *In press.*

Pernot, P., Round, A., Barrett, R., De Maria Antolinos, A., Gobbo, A., Gordon, E., Huet, J., Kieffer, J., Lentini, M., Mattenet, M., *et al.* (2013). Upgraded ESRF BM29 beamline for SAXS on macromolecules in solution. J. Synch. Rad. *20*, 660-664.

Prosser, B.E., Johnson, S., Roversi, P., Herbert, A.P., Blaum, B.S., Tyrrell, J., Jowitt, T.A., Clark, S.J., Tarelli, E., Uhrin, D., *et al.* (2007). Structural basis for complement factor H linked age-related macular degeneration. J. Exp. Med. *204*, 2277-2283.

Raman, E.P., Guvench, O., and MacKerell, A.D., Jr. (2010). CHARMM additive all-atom force field for glycosidic linkages in carbohydrates involving furanoses. J. Phys. Chem. B *114*, 12981-12994.

Rayner, L.E., Hui, G.K., Gor, J., Heenan, R.K., Dalby, P.A., and Perkins, S.J. (2014). The Fab conformations in the solution structure of human immunoglobulin G4 (IgG4) restrict access to its Fc region: implications for functional activity. J. Biol. Chem. *289*, 20740-20756.

Rayner, L.E., Hui, G.K., Gor, J., Heenan, R.K., Dalby, P.A., and Perkins, S.J. (2015). The solution structures of two human IgG1 antibodies show conformational stability and accommodate their C1q and FcgammaR ligands. J. Biol. Chem. *290*, 8420-8438.

Rodriguez, E., Nan, R., Li, K., Gor, J., and Perkins, S.J. (2015). A revised mechanism for the activation of complement C3 to C3b: a molecular explanation of a disease-associated polymorphism. J. Biol. Chem. *290*, 2334-2350.

Round, A., Felisaz, F., Fodinger, L., Gobbo, A., Huet, J., Villard, C., Blanchet, C.E., Pernot, P., McSweeney, S., Roessle, M., *et al.* (2015). BioSAXS Sample Changer: a robotic sample changer for rapid and reliable high-throughput X-ray solution scattering experiments. Acta Cryst. D*71*, 67-75.

Roversi, P., Johnson, S., Caesar, J.J., McLean, F., Leath, K.J., Tsiftsoglou, S.A., Morgan, B.P., Harris, C.L., Sim, R.B., and Lea, S.M. (2011a). Structures of the rat complement regulator CrrY. Acta Cryst. F*67*, 739-743.

Roversi, P., Johnson, S., Caesar, J.J., McLean, F., Leath, K.J., Tsiftsoglou, S.A., Morgan, B.P., Harris, C.L., Sim, R.B., and Lea, S.M. (2011b). Structural basis for complement factor I control and its disease-associated sequence polymorphisms. Proc. Natl. Acad. Sci. USA *108*, 12839-12844.

Sali, A., and Blundell, T.L. (1993). Comparative protein modelling by satisfaction of spatial restraints. J. Mol. Biol. *234*, 779-815.

Saphire, E.O., Parren, P.W., Pantophlet, R., Zwick, M.B., Morris, G.M., Rudd, P.M., Dwek, R.A., Stanfield, R.L., Burton, D.R., and Wilson, I.A. (2001). Crystal structure of a neutralizing human IGG against HIV-1: a template for vaccine design. Science *293*, 1155-1159.

Schuck, P. (1998). Sedimentation analysis of noninteracting and self-associating solutes using numerical solutions to the Lamm equation. Biophys. J. *75*, 1503-1512.

Schuck, P. (2000). Size-distribution analysis of macromolecules by sedimentation velocity ultracentrifugation and lamm equation modeling. Biophys. J. *78*, 1606-1619.

Semenyuk, A.V., and Svergun, D.I. (1991). GNOM – a program package for small-angle scattering data-processing. . J. Appl. Crystallogr. *48*, 953-961.

Skjoedt, M.O., Roversi, P., Hummelshoj, T., Palarasah, Y., Rosbjerg, A., Johnson, S., Lea, S.M., and Garred, P. (2012). Crystal structure and functional characterization of the complement regulator mannose-binding lectin (MBL)/ficolin-associated protein-1 (MAP-1). J. Biol. Chem. *287*, 32913-32921.

Teillet, F., Gaboriaud, C., Lacroix, M., Martin, L., Arlaud, G.J., and Thielens, N.M. (2008). Crystal structure of the CUB1-EGF-CUB2 domain of human MASP-1/3 and identification of its interaction sites with mannan-binding lectin and ficolins. J. Biol. Chem. *283*, 25715-25724.

Wallis, R., and Dodd, R.B. (2000). Interaction of mannose-binding protein with associated serine proteases: effects of naturally occurring mutations. J. Biol. Chem. *275*, 30962-30969.

Wright, D.W., and Perkins, S.J. (2015). SCT: a suite of programs for comparing atomistic models with small-angle scattering data. J. Appl. Cryst. *48*, 953-961.

Wu, J., Wu, Y.Q., Ricklin, D., Janssen, B.J., Lambris, J.D., and Gros, P. (2009). Structure of complement fragment C3b-factor H and implications for host protection by complement regulators. Nat. Immunol. *10*, 728-733.
