# Supplementary material for: Flexibility in Mannan-Binding Lectin-Associated Serine Proteases-1 and -2 Provides Insight on Lectin Pathway Activation
Source: Structure. 2017 Feb 7;25(2):364–75. doi: 10.1016/j.str.2016.12.014 (PMC5300068; doi:10.1016/j.str.2016.12.014)
Supplement: Document S2. Article plus Supplemental Information [file mmc8.pdf]

Article

Structure

Flexibility in Mannan-Binding Lectin-Associated Serine Proteases-1 and -2 Provides Insight on Lectin Pathway Activation

Highlights

- Ca^{2+}-bound MASP crystal structures reveal binding sites for MBL and other ligands
- The MASP crystal structures predict linear domain structures
- Solution structures of MASP-1 and MASP-2 are bent by as much as 90° from linearity
- Flexibility in MASP dimers may permit both intra- and inter-complex activation

Authors

Ruodan Nan (南若丹),
Christopher M. Furze, David W. Wright,
Jayesh Gor, Russell Wallis,
Stephen J. Perkins

Correspondence

s.perkins@ucl.ac.uk

In Brief

Nan et al. show that MASPs, the serine proteases responsible for initiating the lectin pathway of complement activation, are much more flexible in solution than previously thought. This flexibility probably facilitates both intra- and inter-complex activation when mannan-binding lectin-MASP complexes bind to pathogen surfaces.

Nan et al., 2017, Structure *25*, 364–375
February 7, 2017 © 2017 The Authors. Published by Elsevier Ltd.
http://dx.doi.org/10.1016/j.str.2016.12.014

CellPress

Flexibility in Mannan-Binding Lectin-Associated Serine Proteases-1 and -2 Provides Insight on Lectin Pathway Activation

Ruodan Nan (南若丹),[1] Christopher M. Furze,[2] David W. Wright,[1] Jayesh Gor,[1] Russell Wallis,[2] and Stephen J. Perkins[1,3,*]
[1]Department of Structural and Molecular Biology, Division of Biosciences, University College London, Darwin Building, Gower Street, London WC1E 6BT, UK
[2]Departments of Infection, Immunity and Inflammation and Molecular Cell Biology, University of Leicester, University Road, Leicester, LE1 9HN, UK
[3]Lead Contact
*Correspondence: s.perkins@ucl.ac.uk
http://dx.doi.org/10.1016/j.str.2016.12.014

SUMMARY

The lectin pathway of complement is activated by complexes comprising a recognition component (mannose-binding lectin, serum ficolins, collectin-LK or collectin-K1) and a serine protease (MASP-1 or MASP-2). MASP-1 activates MASP-2, and MASP-2 cleaves C4 and C4b-bound C2. To clarify activation, new crystal structures of Ca^{2+}-bound MASP dimers were determined, together with their solution structures from X-ray scattering, analytical ultracentrifugation, and atomistic modeling. Solution structures of the CUB1-EGF-CUB2 dimer of each MASP indicate that the two CUB2 domains were tilted by as much as 90° compared with the crystal structures, indicating considerable flexibility at the EGF-CUB2 junction. Solution structures of the full-length MASP dimers in their zymogen and activated forms revealed similar structures that were much more bent than anticipated from crystal structures. We conclude that MASP-1 and MASP-2 are flexible at multiple sites and that this flexibility may permit both intra- and inter-complex activation.

INTRODUCTION

Complement destroys invading microorganisms and initiates defense mechanisms including chemotaxis, phagocytosis, cell adhesion, B cell differentiation, and maintenance of immune tolerance (Carroll, 2004; Porter and Reid, 1978). It also facilitates tissue remodeling, homeostasis, and resolution of inflammation via apoptosis and clearance of cellular debris (Botto, 1998; Taylor et al., 2000). Defects in complement are associated with immunodeficiencies, autoimmune diseases, tissue damage, anaphylaxis, transplant rejection, and necrosis. Complement via the lectin pathway is initiated when mannan-binding lectin (MBL), serum ficolins, collectin-KL, and/or collectin-K1 bind to pathogens and activate MBL-associated serine proteases (MASP-1 and MASP-2 and MASP-3) (Figure 1). The importance of the lectin pathway is highlighted by common immunodeficiencies associated with mutations to MBL (Turner, 1996) and MASP-2 (Stengaard-Pedersen et al., 2003). Inappropriate lectin pathway activation causes tissue damage following transient ischemia (Schwaeble et al., 2011), so modulators of the lectin pathway are likely to have important therapeutic benefits.

MBLs are large oligomers (150–300 kDa), predominantly dimers to tetramers of a homotrimeric subunit (Wallis and Drickamer, 1999). In each homotrimer, pathogen recognition is mediated through the coordinated binding of three globular carbohydrate recognition domains (CRDs) connected to a rod-like collagenous stem (Figure 1). MASPs are homodimers that circulate as zymogens bound to the collagenous domains of MBL (Wallis and Dodd, 2000). Each MASP comprises two N-terminal CUB (C1r/C1s, Uegf, and bone morphogenetic protein-1) domains separated by an epidermal growth factor (EGF)-like domain followed by two short complement regulator (SCR) domains and a C-terminal serine protease (SP) domain (Figure 1). MASP-1 and MASP-3 are alternatively spliced products from the same gene. Activation occurs following cleavage at a single site between the SCR2 and SP domains when MBLs bind to a pathogen surface. MASP-1 cleaves MASP-2, which subsequently cleaves the downstream complement components C4 and C4-bound C2 to form the C3 convertase (Wallis et al., 2007). MASP-1 and/or MASP-3 facilitate activation of the alternative pathway through activation of factors D and B (Iwaki et al., 2011; Takahashi et al., 2010). It is often assumed that the MBLs adopt bouquet-like structures similar to complement C1q. Instead, our recent atomistic scattering modeling showed that they have near-planar, fan-shaped structures in solution (Figure 1), which raises new questions on how the complexes activate complement (Miller et al., 2012). In two different theories following attachment of the MBL-MASP complex to a pathogenic surface, either (1) each monomer within the MASP dimer is able to bend significantly to enable one SP domain to activate its partner, as occurs for the MASP homologs, C1r and C1s within the C1 complex, or (2) a given MASP-MBL complex activates neighboring MASP molecules on adjacent MBL-MASP complexes (Degn et al., 2014; Wallis et al., 2010). A recent analysis by small- angle X-ray scattering (SAXS) and electron microscopy (EM) favored the inter-complex model, concluding that MASP-1 was only modestly flexible and the protease domains

 © 2017 The Authors. Published by Elsevier Ltd.
This is an open access article under the CC BY license (http://creativecommons.org/licenses/by/4.0/).

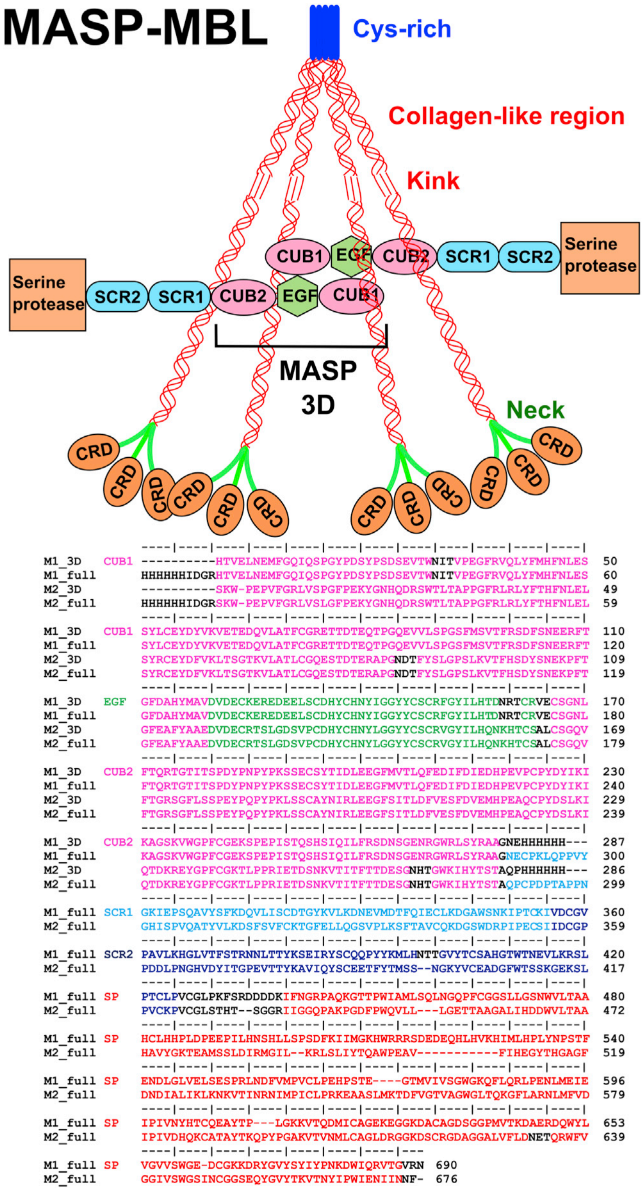

Figure 1. Domain Organization of MASP and Its MASP-MBL Complex

Each MASP monomer is composed of six domains. An MBL monomer is a homotrimer of three polypeptides, each forming an N-terminal cysteine-rich region, a collagen-like region, an α-helical neck region, and a C-terminal carbohydrate recognition domain (CRD). An interruption ("kink") may induce flexibility in the collagen region. A schematic MBL tetramer is shown bound to an MASP dimer at two CUB1 domains. The MASP sequences are colored according to the MASP domains. Glycosylation sites are shown in black, together with expression tags and linkers.

were too far apart to enable intra-complex activation (Kjaer et al., 2015).

Here, we investigate the dimer structures of MASP-1 and MASP-2 through the combination of SAXS, analytical ultracentrifugation (AUC), and a new atomistic modeling procedure (Perkins et al., 2011, 2016). While several crystal structures are known for the N-terminal MASP fragments (Feinberg et al., 2003; Gingras et al., 2011; Gregory et al., 2004; Skjoedt et al., 2012; Teillet et al., 2006) and for the C-terminal MASP fragments (Dobo et al., 2009; Gal et al., 2005; Harmat et al., 2004; Heja et al., 2012; Kidmose et al., 2012), these are incomplete. In particular, MASP-2 CUB1-EGF-CUB2 (subsequently called MASP-2 3D) was crystallized in the presence of citrate that chelates Ca^{2+}, and although Ca^{2+} was bound to the EGF-like domain, the CUB1 and CUB2 domains lacked Ca^{2+} and became partially disordered as a result (Feinberg et al., 2003). Here, to complete our knowledge of the MASP structures, we first applied X-ray crystallography to the N-terminal fragments to identify the importance of bound Ca^{2+}. Next, by applying our strategy to the zymogen and activated forms of MASP, we assessed whether the MASP dimer is sufficiently flexible to allow auto-activation or inter-molecular activation.

There are four potential binding sites for MBL stems on each MASP homodimer—one on each of the four CUB domains (Wallis and Dodd, 2000; Figure 1). The crystal structures of collagen-like peptides bound to CUB1 and CUB2 mean we now know the MBL-MASP contacts at atomic resolution (Gingras et al., 2011; Venkatraman Girija et al., 2013). These structures reveal key contacts between an essential Lys46 residue in the collagen-like region of MBL and three Ca^{2+}-binding residues in CUB2. The Ca^{2+}-binding site is conserved in CUB1, which binds to a separate collagen stem. By comparing the MASP solution structures with models for MBL, we evaluated the MASP-MBL complexes that trigger complement activation. In particular, our new MASP structures clarify the extent to which the MASPs undergo significant conformational changes during activation after pathogen binding by MBL.

RESULTS

Purification and Crystal Structure Determination of MASPs

Following the crystallography and scattering strategy used for rat MBL (Miller et al., 2012), we expressed full-length rat MASP-1 and MASP-2 zymogens and both their 3D fragments for structural analysis (Figure 1). Repeated attempts to obtain diffracting crystals of full-length MASP-1 and MASP-2 were unsuccessful, probably due to inter-domain flexibility. An alternative strategy involving crystallography of MASP fragments was followed. Because there was no structure of rat MASP-1 3D, and the existing structure of rat MASP-2 3D lacked two of the three Ca^{2+}, including those Ca^{2+} that form part of the binding site for MBL, both rat MASP-1 3D and MASP-2 3D were crystallized in the presence of 2 mM calcium and diffraction data were collected. This resulted in one MASP-1 structure at 3.7 Å resolution and three independent MASP-2 structures at 2.6–2.7 Å resolution (Table 1).

The four electron density maps were of high quality (Figure S1). For MASP-1 3D, 276 of its 281 residues could be built into the electron density map. The resulting structure was similar to the corresponding structure of human MASP-1 (PDB: 3DEM), with a root-mean-square deviation (RMSD) of 0.961 Å over 267 residues (Teillet et al., 2008). For MASP-2 3D, 279 of 280 residues were visible. Interestingly, the three structures overlaid closely, with RMSDs of 0.682, 0.871, and 0.992 Å over 276 Cα atoms. The only notable differences were in the N-terminal seven

Table 1. Crystallographic Data Collection and Refinement Statistics for Rat MASP-2 3D and MASP-1 3D

	MASP-2 MPD/Acetate	MASP-2 PEG 20K	MASP-2 MPD/Citrate	MASP-1
Data Collection				
Beamline	Diamond I04-1	Diamond I04-1	Cu^{2+} home source	Diamond I04
Space group	P 2_1 2_1 2_1	C 2 2 2_1	C 2 2 2_1	I 2 3
a, b, c (Å)	83.6, 91.3, 127.1	66.9, 98.3, 121.4	82.9, 119.4, 104.0	152.7, 152.7, 152.7
α, β, γ, °	90, 90, 90	90, 90, 90	90, 90, 90	90, 90, 90
Resolution (Å)	63.6–2.6 (2.69–2.60)	50.3–2.58 (2.60–2.58)	22.9–2.73 (2.82–2.73)	76.4–3.70 (4.14–3.70)
R_{sym}	0.082 (0.321)	0.099 (0.645)	0.167 (0.653)	0.082 (0.743)
$I/\sigma(I)$	16.04 (2.10)	11.55 (2.03)	6.4 (1.53)	9.2 (1.51)
Completeness	97.6 (98.8)	97.6 (93.5)	99.4 (94.4)	99.9 (99.9)
Redundancy	5.0 (5.2)	3.9 (3.6)	2.0 (2.0)	5.6 (5.5)
Refinement				
Resolution (Å)	63.55–2.6 (2.69–2.60)	50.34–2.58 (2.67–2.58)	22.9–2.73 (2.82–2.73)	76.4–3.70 (3.84–3.70)
No. of reflections	29,893 (2,998)	12,626 (1,195)	14,031 (1,319)	6,473 (1827)
R_{work}/R_{free}	0.205/0.254	0.207/0.245	0.204/0.266	0.248/0.294
No. of atoms	4,502	2,303	2,369	2,271
Protein	4,433	2,208	2,211	2,239
Ligand/ion	6	17	17	32
Water	63	78	141	–
B factors (Å^2)	93.40	51.60	34.70	187.20
Protein	93.90	51.70	35.00	187.60
Ligand/ion	65.80	78.20	65.90	155.50
Water	62.30	44.30	26.80	–
PDB	5CKN	5CIS	5CKM	5CKQ
RMSDs				
Bond lengths (Å)	0.004	0.003	0.004	0.002
Bond angles (°)	1.01	0.72	0.82	0.75
Ramachandran (%)				
Favored	94	93	94	92
Allowed	6	7	6	8
Disallowed	0	0	0	0

The buffers are defined in full in the Supplemental Information. The highest resolution shell is shown in parentheses.

residues and in a loop within the EGF-like domain (Thr125–Ser130), probably reflecting flexibility. All three MASP-2 3D structures were similar to the previous structure of rat MASP-2 3D (PDB: 1NT0) with RMSDs of 1.20, 1.35, and 1.57 Å over 257 Cα atoms (Feinberg et al., 2003). A major difference, however, was that the structures now contained three bound Ca^{2+} compared with the single Ca^{2+} in the EGF-like domain of the earlier MASP-2 3D structure. Each CUB domain bound one Ca^{2+}, where the Ca^{2+}-binding residues and binding loops were well defined. These Ca^{2+} sites are functionally important because they orientate and form the binding site for MBL, ficolins, CL-KL, and CL-K1 (Gingras et al., 2011). All four crystal structures revealed a dimer of MASP-1 3D or MASP-2 3D, formed by antiparallel neighboring contacts between pairs of CUB1-EGF domains about the crystallographic symmetry axis (Figures 2A and 2B). The same dimer was seen in earlier crystal structures of MASPs (PDB: 1NT0 and 3DEM), further supporting earlier conclusions that this is physiologically relevant (Feinberg et al., 2003; Gregory et al., 2003; Skjoedt et al., 2012; Teillet et al., 2008). The total buried surface area for MASP-1 at the dimer interface was 880 Å^2 per molecule (1,760 Å^2 in total), whereas the three crystal structures for MASP-2 gave 1,200, 1,090, and 950 Å^2 per molecule. These values were in excess of 800 Å^2, consistent with stable dimer formation (Rupp, 2010).

Bioavailable calcium is typically present in plasma at around 2.5 mM (Hurwitz, 1996). All four new crystal structures revealed well-ordered, similar calcium-binding sites in each of the CUB1 and CUB2 domains (Figures 2A and 2B). These were previously seen for human MASP-1 3D, but not for rat MASP-2 3D (Figures 2C and 2D). In the MASP-1 CUB1 domain, Ca^{2+} formed contacts with six oxygen atoms in the side chains of Glu49, Asp57, Asp102, Ser104, and Asn105 (distances between 2.3 and 2.4 Å). In the MASP-2 CUB1 domain, Ca^{2+} formed contacts with six oxygen atoms in the side chains of Glu48, Asp56, Asp101, and Asn104 and the main chain oxygen of Ser103 (distances between 2.3 and 2.7 Å; Figure S1A). In the MASP-1 CUB2 domain, Ca^{2+} now formed contacts with five oxygen atoms in the structurally equivalent side chains of Glu216, Asp226, Asp263, and Ser265. In the MASP-2 CUB2 domain, Ca^{2+} formed contacts with five oxygen atoms at the side chains of Glu215, Asp225,

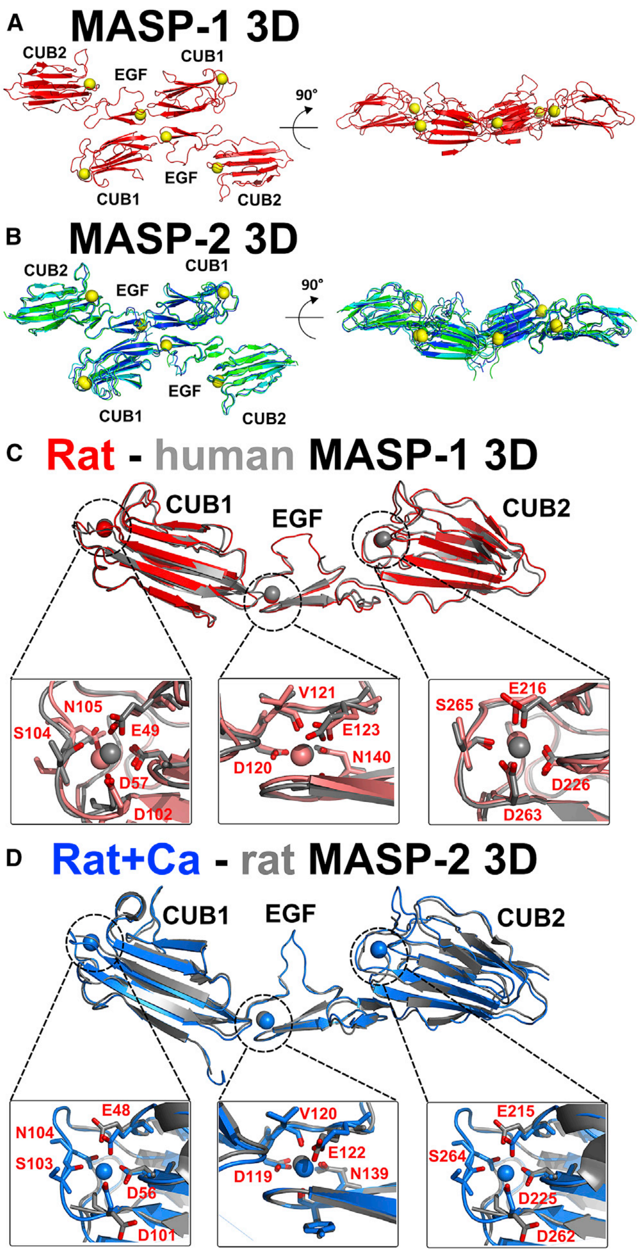

Figure 2. Crystal Structures of MASP-1 and MASP-2 3D Dimers and Their Ca^{2+}-Binding Sites

(A) MASP-1 3D is shown in face-on and side-on views, related by a 90° rotation. Ca^{2+} are shown as yellow spheres.

(B) The three structures for MASP-2 3D are in blue, green, and cyan, also shown in face-on and side-on views.

(C) The rat MASP-1 3D structure (red) is superimposed upon its human equivalent (gray; PDB: 3DEM). All three Ca^{2+} are visible in both structures, with the Ca^{2+}-binding residues shown in red.

(D) The rat MASP-2 3D structure (blue) is superimposed upon its rat equivalent from 2003 (gray; PDB: 1NT0). All three Ca^{2+} are now visible in the new structure, again with Ca^{2+}-binding residues shown in red.

Asp262, and Ser264 (Figure S1B). This second site differed from that in CUB1 only by the omission of the side chain with the structurally equivalent Asn105/Asn104 oxygen atom in CUB2. Nonetheless, this Ca^{2+} site was similar to that reported in the CUB2 domain of MASP-1 alone (Gingras et al., 2011). Comparison of the same CUB1/CUB2 region in the earlier crystal structure of rat MASP-2 showed disorganized binding loops in the absence of calcium (Figure 2D), suggesting that Ca^{2+} stabilizes the MBL-binding residues. The EGF domains of MASP-1 and MASP-2 also bound calcium (Figures 2C and 2D).

X-Ray Scattering of Rat MASPs

X-ray scattering measures by diffraction the overall structure of biological molecules in randomized orientations in solution (Perkins et al., 2011). Here, solution scattering identified the domain structures of MASP-1 3D, MASP-2 3D, MASP-1 zymogen/activated, and MASP-2 zymogen/activated, with and without Ca^{2+}, in order to verify the structures seen by crystallography. The scattering data $I(Q)$ were collected at concentrations dilute enough to minimize concentration effects (0.25–1.36 mg/mL). The MASPs showed no radiation damage or X-ray induced aggregation in the time frame analyses. The time-averaged runs were thus used for data analyses. Guinier analyses of the $I(Q)$ data in two separate Q ranges gave the radius of gyration R_g and the cross-sectional radius of gyration R_{xs} (Figure 3).

The R_g value monitors the overall degree of elongation of the MASP dimers. At the lowest Q values, the R_g values were measured within satisfactory $Q.R_g$ limits in concentration series between 0.25 and 1.26 mg/mL for the six MASPs (Figure 3A). The mean of two to four R_g values were 3.83 ± 0.02 nm for MASP 3D and 7.73 ± 0.20 nm for full-length MASP (Table 2). The R_g values for the full-length MASPs were almost double those of the MASP 3Ds, this being as expected if the full-length MASPs have extended six-domain structures. No change with protein concentrations in either the $I(0)/c$ or R_g values for each of the six MASPs was observed. This meant that no self-association or conformational change in MASP was detectable with change in protein concentration. No change between the pairs of R_g values for zymogen and activated MASP-1 and MASP-2 was seen (Table 2), indicating that their domain arrangements were unaffected by cleavage of the SCR2-SP linker.

The cross-sectional R_{xs} Guinier analyses monitored the shorter dimensions of the MASP dimers. The ln $I(Q).Q$ versus Q^2 plots showed linear regions in the 3D and full-length proteins (Figure 3B), indicating that both proteins were elongated. The resulting R_{xs} values for MASP-1 3D and MASP-2 3D were similar at 1.94 ± 0.01 nm and 1.74 ± 0.02 nm, respectively (Table 2). For the full-length zymogen and activated MASP, the four $I(Q).Q$ versus Q^2 plots showed inflexions before a linear R_{xs} region, indicating complex but similar domain arrangements in MASPs. The resulting R_{xs} values were indistinguishable for zymogen and activated MASP-1 (at 1.53–1.54 nm) and MASP-2 (both at 1.46 nm). This indicated no major conformational differences between the zymogen and activated forms, even with the addition or removal of Ca^{2+}. The similar R_{xs} values for MASP-1 and MASP-2 indicated that both showed similar cross-sectional structures.

The distance distribution function $P(r)$ represented all the distances between pairs of atoms within the macromolecule. This provided structural information in real space. The $P(r)$ curve provided an alternative calculation of the Guinier R_g value and gave maximum lengths (L) following an assumption of the maximum dimension (D_{max}). The R_g values from $P(r)$ were similar to those

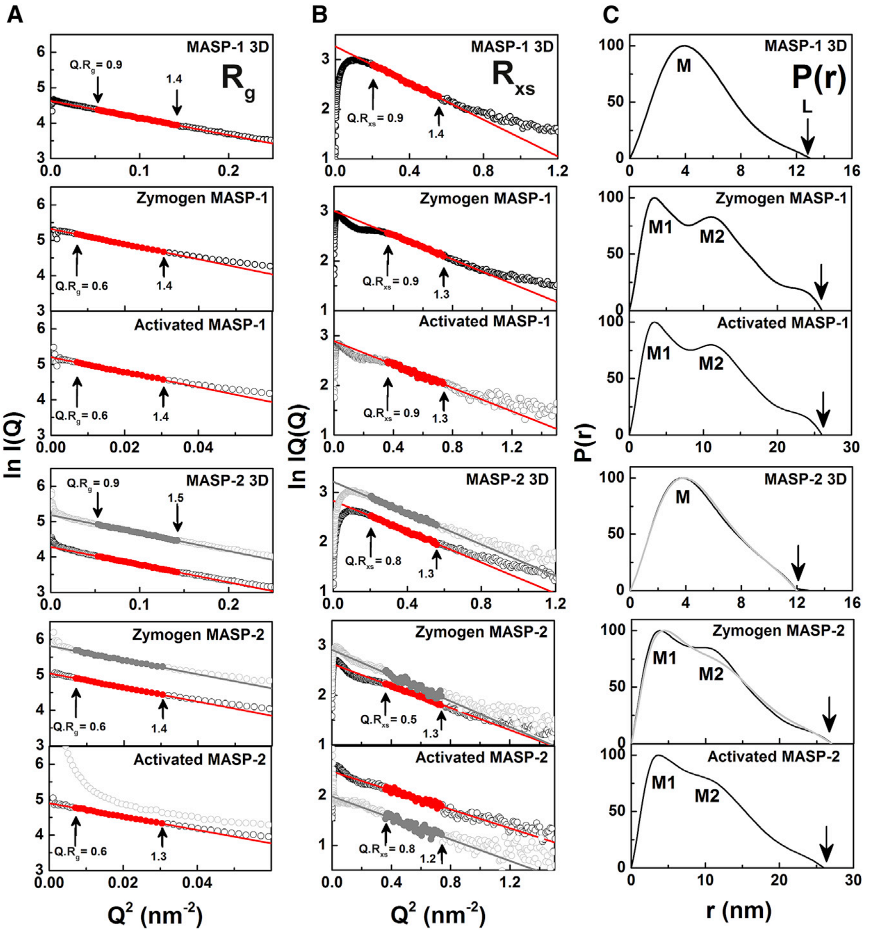

Figure 3. SAXS Guinier and $P(r)$ Analyses

The upper three rows correspond to MASP-1 with Ca^{2+}. The bottom three rows correspond to MASP-2 with (gray) and without Ca^{2+} (black). The R_g, R_{xs}, and $P(r)$ analyses for each protein are shown.

(A and B) Open circles correspond to data points, and filled circles correspond to those used for the Guinier R_g and R_{xs} values. The Q ranges for the R_g and R_{xs} fits are arrowed (Supplemental Information).

(C) The $P(r)$ curves were normalized to 100 for clarity. The maximum length of each molecule is denoted by L at the r values where the $P(r)$ curves reach 0. The peak maxima M for the MASP 3D and $M1$ and $M2$ for MASP-1 and MASP-2 represent the most frequent distances within their structures.

from the Guinier analyses as expected (Table 2). The maximum length L of the MASPs was determined from the r value when $P(r) = 0$ at large r (Figure 4C). For both 3D MASPs, a single peak was observed in $P(r)$ with the most common intra-particle distance at a maximum M of 3.9 nm and a length L of 13 nm. This M value corresponds well to the mean intra-domain distance within the four CUB domains. For zymogen and activated full-length MASP, two peaks $M1$ and $M2$ at 3.4 nm and 10.7 nm, respectively, were seen. $M1$ was assigned to structures similar to those in MASP 3D. $M2$ corresponded well to the distances between the two SP domains and the CUB domains in the center of the MASP dimer.

Analytical Ultracentrifugation of Rat MASPs

AUC studies solution structures by monitoring their sedimentation under high centrifugal force (Cole et al., 2008). This provided information on the structure and oligomerization of MASP. The latter was important because the MASPs were purified in low 20 mM NaCl salt, which may promote oligomer formation. Sedimentation velocity experiments were performed on the six MASPs at concentrations similar to those used for SAXS in physiological ionic strength conditions in the same buffers with Ca^{2+} or EDTA (Figures 4A and 4E). The velocity data were analyzed using absorbance optics to generate the size distribution analyses $c(s)$. The analyses of up to 80 boundaries revealed excellent fits and satisfactory RMSDs. The mass determination from the single $c(s)$ peak showed that all six samples were dimers with no detectable monomer or higher oligomer (Figures 4B–4D and 4F–4H). The molecular mass of MASP-1 3D was 74 ± 2 kDa, and that for MASP-2 3D was 65 ± 2 kDa, in good agreement with sequence-calculated masses of 75 kDa for MASP-1 3D and 74 kDa for MASP-2 3D. That for zymogen MASP-1 was 163 ± 6 kDa, activated MASP-1 was 158 ± 6 kDa, zymogen MASP-2 was 147 ± 6 kDa, and activated MASP-2 was 166 ± 1 kDa. These values agreed well with the sequence-predicted masses of 170 kDa and 163 kDa for full-length MASP-1 and MASP-2 dimers, respectively. The sedimentation coefficient $s_{20,w}$ provided an independent measure of macromolecular elongation. The $s_{20,w}$ values of MASP-1 3D and MASP-2 3D were 4.53 S and 4.09 S, respectively (Table 2). The $s_{20,w}$ values of zymogen and activated MASP-1 were 5.90 S and 5.79 S, respectively, and those for zymogen and activated MASP-2 were 5.42 S and 5.62 S, respectively (Table 2). The $s_{20,w}$ value for MASP-2 3D was similar to that of 4.55 ± 0.10 S reported previously (Feinberg et al., 2003).

The availability of initial models or structures for MASP permitted comparison of the experimental $s_{20,w}$ values with those predicted from these. The precision of the comparisons is ±0.21 S (Perkins et al., 2016). Interestingly, the prediction of 4.26 S from the MASP-1 3D crystal structure was similar to the observed value of 4.53 ± 0.08, while that of 4.18 S from the MASP-2 crystal structure was close to the observed value of 4.09 ± 0.07 S (Table 2). The solution structures of both proteins thus broadly resembled those seen by crystallography (Figures 2A and 2B). In distinction, for the zymogen and activated forms of MASP-1 and MASP-2, these comparisons showed deviations from the initial models. For the full-length MASPs, the initial predictions of 5.38 S, 5.50 S, 5.28 S, and 5.28 S (Figures 4C, 4D, 4G, and 4H) were lower than the observed values of 5.90 ± 0.15 S, 5.79 ± 0.13 S, 5.42 ± 0.15 S, and 5.62 ± 0.16 S, respectively (Table 2), indicating that the solution structures were more bent than the initial extended

Table 2. Experimental and Modeled X-Ray Scattering and Sedimentation Coefficient Data

	R_g (nm)[a]	R_{xs} (nm)	L (nm)	$s_{20,w}$ (S)[b]
Experimental				
MASP-1 3D (12 curves)	3.79 ± 0.01<br>3.97 ± 0.01	1.94 ± 0.01	13	4.53 ± 0.08
MASP-2 3D (9 curves)	3.87 ± 0.02<br>3.99 ± 0.01	1.74 ± 0.02	12	4.09 ± 0.07
Zymogen MASP-1 (14 curves)	7.93 ± 0.09<br>8.23 ± 0.02	1.54 ± 0.02	26	5.90 ± 0.15
Activated MASP-1 (8 curves)	7.86 ± 0.12<br>8.22 ± 0.02	1.53 ± 0.02	26	5.79 ± 0.13
Zymogen MASP-2 (9 curves)	7.58 ± 0.13<br>7.98 ± 0.02	1.46 ± 0.06	27	5.42 ± 0.15
Activated MASP-2 (4 curves)	7.54 ± 0.04<br>7.79 ± 0.04	1.46 ± 0.05	26	5.62 ± 0.16
Best-Fit Models				
MASP-1 3D	3.79 ± 0.09	1.93 ± 0.03	14.1 ± 0.7	4.25 ± 0.07
MASP-2 3D	3.92 ± 0.07	1.89 ± 0.33	14.3 ± 0.5	3.96 ± 0.03
Zymogen MASP-1	7.73 ± 0.17	1.71 ± 0.06	26.4 ± 0.9	5.92 ± 0.05
Activated MASP-1	8.09 ± 0.23	1.67 ± 0.10	25.1 ± 1.2	5.92 ± 0.07
Zymogen MASP-2	7.34 ± 0.27	1.32 ± 0.22	23.5 ± 1.0	5.69 ± 0.10
Activated MASP-2	7.70 ± 0.18	1.49 ± 0.10	25.3 ± 1.1	5.61 ± 0.10

[a]The mean R_g values from the Guinier analyses are shown first, followed by the mean R_g values from the $P(r)$ analyses, the mean R_{xs} values from the Guinier analyses, and the mean L values from the $P(r)$ analyses (Figure 3).

[b]The mean $s_{20,w}$ values observed at 50,000 rpm are shown (Figure 4).

homology models. This evidence for more compact structures indicated flexibility in the MASP-1 and MASP-2 structures that had not been previously recognized.

Atomistic Scattering Modeling of Rat MASPs

As for rat MBL (Miller et al., 2012), atomistic modeling for the six MASPs assessed whether the two linear MASP 3D crystal structures and four linear homology models for full-length MASP agreed with the experimental SAXS data. The predicted R_g values of 3.62 nm and 3.58 nm from the MASP-1 3D and MASP-2 3D crystal structures were less than but similar to the observed R_g values of 3.79 ± 0.01 nm and 3.87 ± 0.02 nm (Table 2; Figures 5A and 5D) to indicate that their solution structures broadly resembled their crystal structures (Figures 2A and 2B). In contrast, the linear homology models of zymogen and activated full-length MASP-1 gave predicted R_g values of 9.00 nm and 8.68 nm that were 10–13% greater than the observed R_g values of 7.93 ± 0.09 nm and 7.86 ± 0.12 nm (Table 2; Figures 5B and 5C). The predicted R_g values of 8.36 nm and 8.23 nm for the initial models of zymogen and activated full-length MASP-2 were likewise 9–10% greater than the observed R_g values of 7.58 ± 0.13 nm and 7.54 ± 0.04 nm (Table 2; Figures 5E and 5F). These R_g differences show that the full-length proteins in solution were more bent and compact in shape than the linear initial models, in agreement with the modeling of the $s_{20,w}$ values.

To identify detailed 3D solution structures, atomistic modeling searches were performed (Perkins et al., 2016). The MASP 3D crystal structures were used to generate physically realistic structures. Regions of flexibility were assigned to the EGF-CUB2 linkers and C-terminal hexaHis tags (Figure 1). The CUB1-EGF dimer contacts did not allow flexibility at the CUB1-EGF linker. Using a Monte Carlo procedure, the EGF-CUB2 linker peptides were randomized to generate 1,982–4,517 trial MASP 3D structures for computing their SAXS curves. A large improvement in the goodness-of-fit R factor to 4.8%–7.4% was obtained, compared with 9.2%–11.2% for the two crystal structures (Figures 5A and 5D). This decrease showed that the use of flexible EGF-CUB2 linkers resulted in significantly improved curve fits (Figures 6A and 6D). When two N-glycan oligosaccharides in extended conformations were added to the structures, the agreement between the experimental and calculated $I(Q)$ and $P(r)$ curves improved to R factors of 4.1%–4.2% (Figures 6A and 6D). All ten best-fit structures retain this conformational feature, being bent inward toward the center of the MASP 3D dimer (Figures 7A and 7D). When the degree of bending was parameterized (Figure S2A), the separation of 9.8–10.0 nm between the centers of the CUB2 domains in the two crystal structures was almost unchanged at 9.5 ± 0.5 nm in the ten best-fit models for MASP-1 3D and MASP-2 3D, while the angle between the two CUB2 domains was significantly decreased from 171° to as low as 142 ± 8° (Table S1). As an independent check of the modeling, the predicted $s_{20,w}$ values from the ten best-fit models for each of MASP-1 3D and MASP-2 3D gave mean values of 4.25 ± 0.07 S and 3.96 ± 0.03 S. These were similar to the experimental $s_{20,w}$ values of 4.53 ± 0.08 S and 4.09 ± 0.07 S, as for the two crystal structures (Figures 2A and 2B). In conclusion, while our atomistic modeling revealed similar extended MASP 3D structures in solution and in the crystal, the final SAXS models indicated large differences in the two 3D structures in which the CUB2 domains were bent right back, as much as vertically upward from the plane of the CUB1-EGF domains (Figures 6A, 6D, 7A, and 7D). Importantly for function, these differences suggest that the EGF-CUB2 junction is a flexible hinge with a 90° flex (Figure S2 and Table S1).

Atomistic SAXS modeling was also performed for full-length MASPs using the linear homology models to generate physically realistic varied structures. Flexible regions were assigned at the EGF-CUB2, CUB2-SCR1, SCR1-SCR2, and SCR2-SP linkers and the N-terminal hexaHis tags. The MASP 3D crystal structure was used in this. By a Monte Carlo procedure, the five linker peptides were varied to generate 6,173–30,910 trial MASP structures. The resulting goodness-of-fit R factors versus R_g values (Figures 5B, 5C, 5E, and 5F) showed that the linear homology model R factor values of 7.7%–9.1% were reduced to 6.5%–7.1% for the best-fit models. In addition, the best-fit models showed better agreements between the experimental and modeled R_g values. Bends in the four inter-domain linkers thus

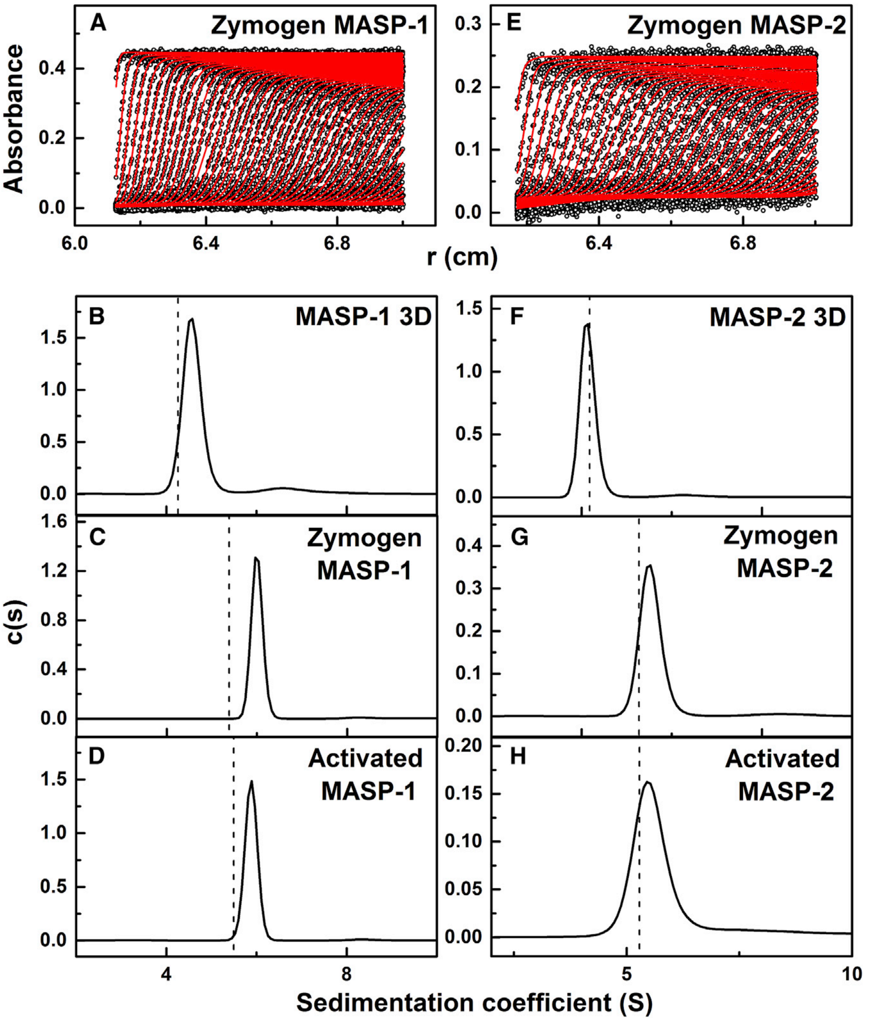

Figure 4. Sedimentation Velocity Analyses of the MASPs

(A and E) Boundary fits for the zymogen of MASP-1 (0.28 mg/mL) and MASP-2 (0.14 mg/mL). Each of the 60 absorbance scans were fitted. The absorbance data are in black, whereas the boundary fits are in red.

(B–H) The six *c*(*s*) distribution analyses are shown for each protein, from which the peaks give the $s_{20,w}$ values. The $s_{20,w}$ values calculated from the crystal and initial homology structures using HYDROPRO are shown as dashed lines.

resulted in improved curve fits (Figures 6B, 6C, 6E, and 6F). When three N-glycosylation oligosaccharides were added to the MASP structures (Figure 1), the *R* factors were improved further to 4.6%–5.2% (Figures 6B, 6C, 6E, and 6F). Increasing the number of trial models showed no further improvement in *R* factors. A control Search 2 started from the best-fit scattering models for MASP 3D and not their crystal structures and gave unchanged *R* factors of 4.2%–5.8% (Figures 5B, 5C, 5E, 5F, 6B, 6C, 6E, and 6F). In summary, the ten best-fit scattering structures showed that the SP domains at the extremities of the dimer move toward each other to create bent structures (Figures 7B, 7C, 7E, and 7F).

The extent of bend in the final full-length MASP-1 and MASP-2 models was parameterized (Figure S2B). The initial separation of 27–29 nm (MASP-1) and 24–25 nm (MASP-2) between the SP domains in the starting models was reduced to 20–22 nm (MASP-1) and 16–20 nm (MASP-2) in the ten best-fit models (Table S1). While the two zymogens may be more compact than the two activated MASPs, any difference was small. The $\theta3$ angles between the two SP domains decreased from 166°–172° in the starting models to 100°–142° (Table S1). As an independent check of the modeling, the predicted $s_{20,w}$ values from the ten best-fit models for zymogen and activated MASP-1 gave mean values of 5.92 S and 5.92 S, and those for zymogen MASP-2 and activated MASP-2 were 5.69 S and 5.61 S. These agreed better with the experimental $s_{20,w}$ value of 5.90 S, 5.79 S, 5.42 S, and 5.62 S in that order (Table 2) than the initial predictions (see above). In conclusion, we have demonstrated bent and flexible MASP-1 and MASP-2 structures in which the two SP domains are closer together.

DISCUSSION

Our crystal structures and X-ray scattering data for six rat MASPs revealed a novel understanding of their solution structures and in particular on their flexibility. We report new experimental structures for intact full-length MASP-1 and MASP-2 and the first identification of flexible junctions between the MASP domains. The SAXS data were supported by ultracentrifugation and atomistic scattering modeling, showing that the full-length MASP solution structures are significantly bent. The EGF-CUB2 junction appeared highly flexible with up to 90° of flex (Figure 7). The combination of these bent and flexible MASP solution structures with our earlier model for an extended planar and flexible MBL structure (Miller et al., 2012) clarifies how the MASP and MBL structures interact with each other to activate the lectin pathway.

Flexibility of the MASP Domain Structures

The domain flexibility in MASPs is complementary to that deduced previously for MBL. Evidence for MASP flexibility comes from (1) the differences between the experimental sedimentation coefficients and the predictions from the initial linear MASP structures (Figures 4B–4D and 4F–4H) and (2) the large deviations between the initial linear MASP structures and its experimental X-ray scattering curve. Flexibility between CUB2 and SCR1 had previously been proposed in the formation of a "closed" MASP structure in which the two SCR1-SCR2-SP moieties and the CUB1-EGF-CUB2 moiety were hypothesized to form the three sides of a triangular domain arrangement that

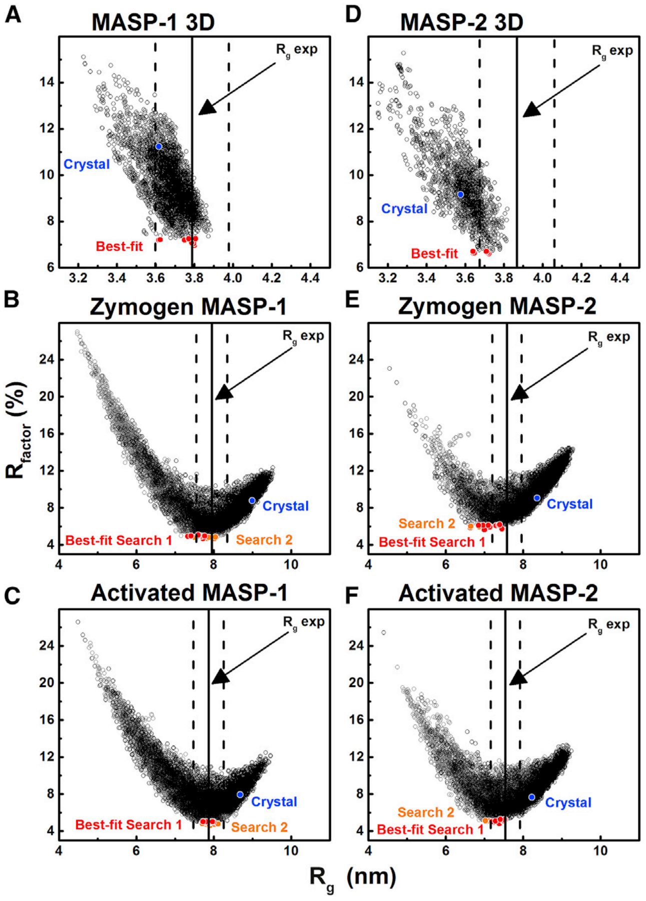

Figure 5. Atomistic Modeling Analyses of the MASPs

(A–F) The *R* factors of the trial models versus their R_g values are shown for each MASP. The ten best-fit models with the lowest *R* factors are in red. For full-length MASP, the best-fit models from Search 2 are in orange. The crystal structure is shown in blue. The experimental R_g values are shown by vertical solid lines (arrowed) with errors of ±5% shown by dashed lines. (A) MASP-1 3D, (B) zymogen MASP-1, (C) activated MASP-1, (D) MASP-2 3D, (E) zymogen MASP-2, (F) activated MASP-2. (A, D) The 4,517 and 1,982 *R* factors are compared with the calculated R_g values for the MASP 3D models. (B, C, E, and F) The 6,173–30,910 *R* factors are compared with the calculated R_g values for the four MASP models.

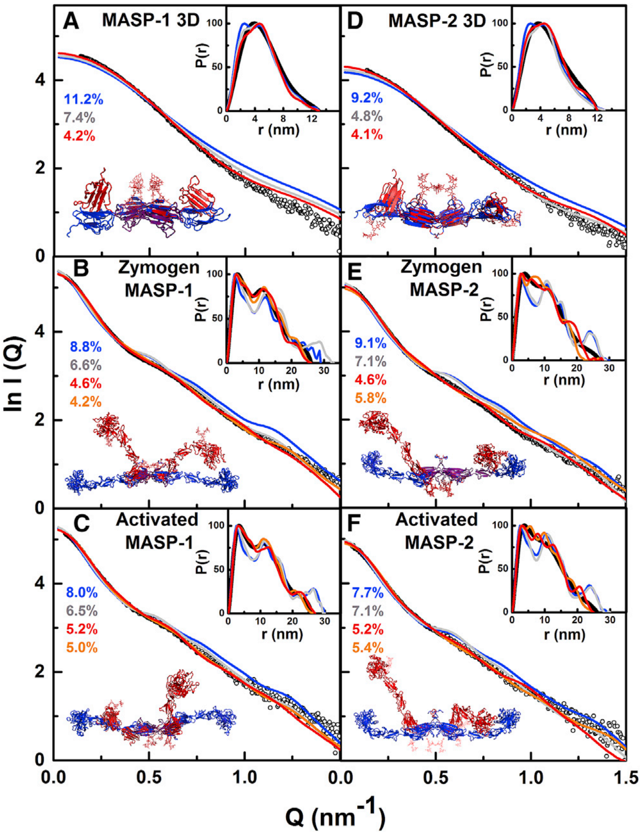

Figure 6. X-Ray Scattering Curve Fits for the Best-Fit MASP Dimer Models

MASP-1 3D (A), zymogen MASP-1 (B), activated MASP-1 (C), MASP-2 3D (D), zymogen MASP-2 (E), activated MASP-2 (F). The experimental curves *I*(*Q*) are shown as black circles. The modeled curves *I*(*Q*) are the colored solid lines with their corresponding *R* factors in the same color. The insets show the experimental (black) and modeled (color) *P*(*r*) curves. (A, D) For MASP-1 3D and MASP-2 3D, the modeled curves for the non-glycosylated starting structures are in blue, the glycosylated starting structures are in gray, and the best-fit structures are in red. The dimer crystal structures for MASP-1 3D and MASP-2 3D are in blue, and the best-fit glycosylated structures are in red. (B, C, E, and F) The modeled curves for the non-glycosylated starting structures are in blue. The curves from the best-fit MASP models from Search 1 are red whereas those from Search 2 are orange. The starting homology dimer models for zymogen or activated MASP-1 or MASP-2 are in blue, and those in red are one of the best-fit MASP dimer models from Search 1. The starting and best-fit structures are superimposed at the CUB1-EGF domains.

enabled the auto-activation of the SP domains (Feinberg et al., 2003; Teillet et al., 2008). Flexibility between the EGF and CUB2 domains was also hypothesized to be part of an activation mechanism for the MASP-MBL complex based on a CUB2-collagen crystal structure (Gingras et al., 2011). Differences of up to 90° between the linear crystal structures and the bent solution structures described here indicate that the EGF-CUB2 junction must be particularly flexible (Figure 7). A previous structural model for full-length MASP-1 based on a crystal structure for the four domains of MAP-1 (Skjoedt et al., 2012) is now seen to be too elongated and rigid when this is compared to the views in Figure 7. MASP flexibility was not considered in detail in a recent SAXS analysis in which 2-fold C2 symmetry was assumed in extended MASP dimer models that were fitted to the data (Kjaer et al., 2015). The current study has now provided clear evidence of MASP flexibility. The importance of bending relates to MASP auto-activation. The extent of bending observed in our best-fit MASP models may permit the two SP domains to come into contact with each other. Although this mechanism is not clearly proven by our present analyses, it cannot be excluded, as was suggested by another recent analysis (Kjaer et al., 2015).

In our previous SAXS structural modeling of MBL, flexibility was deduced from a bend observed between the CRD/neck region and the linear collagen region (Miller et al., 2012). This implies that the 9–12 CRD domains found in MBL trimers and tetramers are able to adjust their orientations and dock to a mannose-coated surface, irrespectively of the roughness of this surface. Each single CRD-mannose interaction has a low

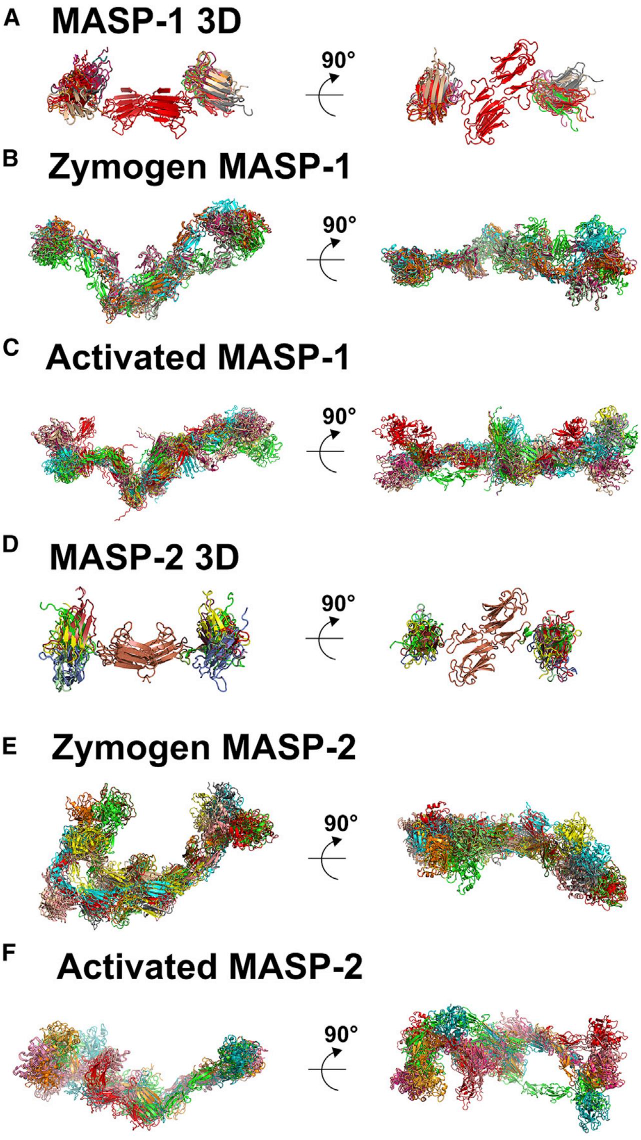

Figure 7. The Best-Fit Solution Structures of the MASP Dimers
MASP-1 3D (A), zymogen MASP-1 (B), activated MASP-1 (C), MASP-2 3D (D), zymogen MASP-2 (E), activated MASP-2 (F). For each protein, the ten best-fit models were aligned based on their steric similarities to show their bent shapes. The views to the right show views that were rotated by 90° about their longest axes.

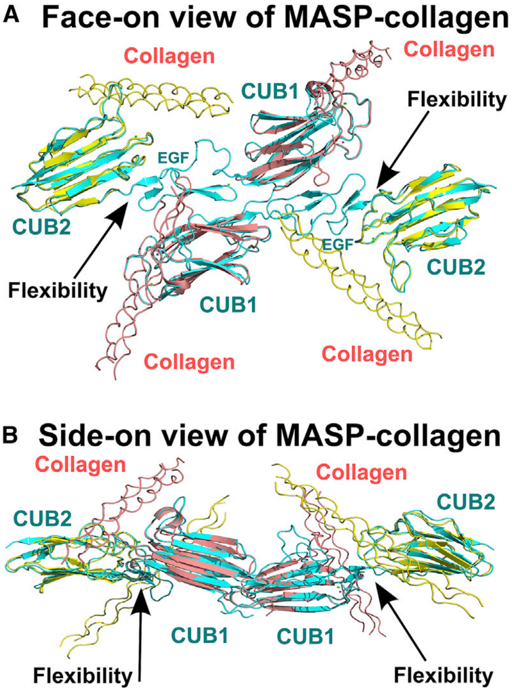

Figure 8. Summary View of the MBL-MASP Complex from Crystallography and Scattering Analyses
Face-on (A) and side-on (B) views of the MBL-MASP complex rotated by 90°. The complex is represented as secondary structure ribbons for four collagen helices bound to MASP-3D at the center. The collagen triple helices bind to the Ca^{2+} sites of the CUB1 and CUB2 domains (Figures 2C and 2D). The flexible regions between the CUB2 and EGF domains are arrowed in black. The MBL-MASP complex was assembled from (1) the complex of the MASP-1 CUB2 domain with collagen (yellow; PDB: 3POB), (2) the C1s 3D complex with a collagen-like peptide from C1q to represent the CUB1 complex (pink; PDB: 4LOR), and (3) one of the three MASP-2 3D crystal structures from this study (cyan; PDB: 5CKN; Table 1).

affinity, with a K_D of about 1 mM; multiple CRD binding increases the affinity by several orders of magnitude.

Complexes between MASP and MBL

Recent crystal structures for the CUB1 and CUB2 domains of C1s and MASP-2 bound to the collagen triple helices of C1q and MBL, respectively, clarify how the MASP-MBL complexes are formed (Gingras et al., 2011; Venkatraman Girija et al., 2013). The superimposition of these two CUB-collagen structures with our MASP-1 and MASP-2 3D crystal structures (Figures 2A and 2B) provides a complete picture of the MASP-MBL complex (Figure 8). The 61-Hyp-Gly-Lys-Leu-Gly-Pro-66 motif within the collagen triple helix of rat MBL forms the binding site for MASP-2 (Wallis et al., 2004). Common to both the CUB1-collagen and CUB2-collagen interactions is Lys46 in the collagen helices, which interacts with three of the residues that coordinate the Ca^{2+} in each CUB domain. The firm identification in this study of the three Ca^{2+} sites in rat MASP-2 now resolves a key ambiguity of the earlier rat MASP-2 structure (Feinberg et al., 2003).

The combination of the MBL and MASP solution structures clarify how the MBL-MASP complex is formed:

(1) Our atomistic solution structures for the MBL dimer, trimer, and tetramer showed near-planar oligomers (Miller et al., 2012). These possess a central hub from which the two, three, or four monomers fan outward and were consistent with EM and atomic force microscopy images of human MBL oligomers (Jensenius et al., 2009), which reported the most common angle between the MBL collagen monomers to be 47 ± 24°. This agreed well with our values of 63° for the dimer; 36° and 6° for the trimer; and 54°, 47°, and 51° for the tetramer (Miller et al., 2012). Because the angle between the collagen helices in our putative MASP-MBL complexes is about 70°

(Figure 8), this angular similarity shows that MBL has a pre-formed collagen conformation that is able to interact with MASP via two of the four potential binding sites. Significant conformational changes of magnitude 10°–20°, however, would be required for simultaneous binding to three or four collagenous stems by the MBL trimers and tetramers.

(2) Our flexible MASP structures revise our current understanding of the activation mechanism. Intra- or inter-molecular MASP mechanisms have been proposed and both have been inferred recently (Degn et al., 2013, 2014); however, there is no direct evidence for either mechanism because the changes that occur when complexes bind to a pathogen surface are not known. In the former, one-half of dimeric rat MASP-2 binds to at least two sites in a near-planar rat MBL structure, and MBL collagen flexibility is reduced when the CRD domains bind to a mannose-coated surface. This may stabilize the MASP-MBL interaction to trigger intra-molecular auto-activation between the two SP domains if they move closer together (Wallis, 2007). In the latter, if the MBL collagen regions form a "bunch-of-tulips" structure similar to that of the hexameric complement C1q structure, as often assumed (Degn et al., 2014), a MASP dimer could bind to all four stems within an MBL collagen cone. The SP domains in MASP will extend outward from such an MBL cone. This binding arrangement is supported by recent EM data of complexes between human MBL tetramers and MASP-1 dimers (Kjaer et al., 2015). That study also favored the inter-complex activation mechanism because it was suggested that MASP-1 polypeptides are not flexible enough to auto-activate based on SAXS and EM data. The findings described here, using SAXS combined with AUC and atomistic modeling, however, contradict this observation and show that both MASPs are much more flexible than previously thought. The observed flexibility suggests that the SP domains could bend toward each other within the complex to lead to intra-molecular MASP auto-activation upon binding to a mannose-coated surface. The choice between an intra- or inter-activation mechanism or a combination of both is presently unclear, in particular because it is not known what happens when MBL-MASP complexes bind to a pathogen surface. Certainly MASP flexibility highlighted by this present study, together with MBL flexibility, means that an intra-molecular MASP auto-activation model cannot be excluded.

(3) Activation does not lead to large structural changes in the MASPs. Comparison of the zymogen and activated forms of MASP-1 and MASP-2 reveals little difference in the conformation or flexibility of MASP-1 or MASP-2. Instead, changes are presumably limited to the protease domain itself, as has been analyzed (Dobo et al., 2009; Gal et al., 2005).

Dimer Interface of the MASP Structures

A crucial issue for MASP function is the stability of the MASP dimer in physiological buffers. Although discussed for MASP crystal structures (Feinberg et al., 2003; Gregory et al., 2004), the observation of a crystallographic dimer does not prove that this dimer exists in solution. In this context, our AUC data showed the complete absence in solution of any monomer peaks at concentrations of 0.8–2.2 μM for full-length MASPs (Figure 4). These data indicate that the dimer dissociation constant K_D will be at least 100-fold less at around 10 nM or less. This is consistent with MASP dimer formation at physiological concentrations of 65–70 nM (MASP-1) and 3–5 nM (MASP-2) in human plasma (Kjaer et al., 2015; Møller-Kristensen et al., 2003; Terai et al., 1997). Thus, the MASP dimers are stable in plasma. Stable dimers are characterized by a buried surface area in excess of 800 Å^2 per molecule (Rupp, 2010). The large buried surface areas of the dimeric 3D crystal structures (Figures 2A and 2B) totaling 880–1,200 Å^2 per molecule fully account for the existence of stable dimers in plasma.

EXPERIMENTAL PROCEDURES

Production, Purification, and Crystallization of MASPs

All six rat MASPs and MASP fragments were produced and purified as described (Chen and Wallis, 2001; Wallis and Dodd, 2000). Rat MASP-1 and MASP-2 and their 3D fragments were produced in Chinese hamster ovary cells (Supplemental Information). The zymogens were enzymatically activated. All six proteins were purified by gel filtration chromatography on a Superdex 200 16/60 column (GE Healthcare) prior to crystallization or further analysis. SDS-PAGE was used to check the purity of all of the samples. The MASP 3D fragments were crystallized by the sitting drop method using the buffers specified in the Supplemental Information. X-ray diffraction data on four sets of crystals for MASP-1 and MASP-2 3D were collected at Leicester on a rotating copper anode source and at Diamond Light Source. Structures were determined by molecular replacement using the crystal structures of human MASP-1 (PDB: 3DEM) and rat MASP-2 (PDB: 1NT0) as search models. Details are provided in the Supplemental Information.

X-Ray Scattering and Analytical Ultracentrifugation

X-ray scattering curves $I(Q)$ for MASP were acquired in two sessions on the BioSAXS robot at Instrument BM29 at the European Synchrotron Radiation Facility, Grenoble, France (Supplemental Information; Pernot et al., 2013; Round et al., 2015). The MASPs showed no radiation damage or X-ray induced aggregation in the time frame analyses. The time-averaged runs were thus used for data analyses to determine the radius of gyration R_g and R_{xs} values from Guinier plots and the distance distribution function $P(r)$ curves. In AUC experiments, sedimentation velocity data were obtained on Beckman XL-I instruments equipped with AnTi50 rotors and using two-sector cells with column heights of 12 mm at a rotor speed of 50,000 rpm. The sedimentation coefficient distribution $c(s)$ analyses fitted the absorbance scans directly to the Lamm equation, in which the sedimentation coefficient was determined from the peak position.

Determination of Solution Structures of MASP-1 and MASP-2

Starting models for all the MASP solution structures were generated from crystal structures. Based on earlier experience of atomistic modeling for dimers, 2-fold symmetry was not assumed for the MASP dimer modeling, and the models sampled both symmetric and asymmetric structures. Each structural model was subjected to two sets of conformational randomization at specified inter-domain linkers to generate broad ranges of trial structures for assessment. Likely flexible linkers between the six individual MASP domains and the His-tag in the starting structures are identified in bold (Figure 1)—two in the 3D proteins and five in the full-length MASPs. A dihedral angle Monte Carlo procedure was used to generate up to 30,000 domain conformations of the six MASPs using SASSIE (Perkins et al., 2016). Sterically overlapping poor models were discarded at their creation. Following this, the scattering curve simulations were performed with SCT (Wright and Perkins, 2015). The output was compared with the experimental X-ray scattering curves by using the

calculated Guinier R_g and R_{xs} values and a goodness-of-fit R factor in order to identify the best-fit solution structures (Supplemental Information). As a further validation of the scattering modeling, sedimentation coefficients $s^o_{20,w}$ for each glycosylated best-fit model were calculated directly from the atomic coordinates using WinHydroPro v1.00, setting the radius of atomic elements as 0.284 nm in order to represent the hydrated structure (Ortega et al., 2011). Full details are described in the Supplemental Information.

SUPPLEMENTAL INFORMATION

Supplemental Information includes Supplemental Experimental Procedures, two figures, one table, and six pdb files and can be found with this article online at http://dx.doi.org/10.1016/j.str.2016.12.014.

AUTHOR CONTRIBUTIONS

R.N. and R.W. designed and conducted the experiments, analyzed data, and contributed toward the writing. C.M.F. and J.G. conducted the experiments. R.N. and D.W.W. performed the modeling. S.J.P. designed and analyzed the experiments and wrote the manuscript. All authors edited the manuscript.

ACKNOWLEDGMENTS

We thank Dr. Martha Brennich, Dr. Petra Pernot, and Dr. Adam Round at the ESRF in Grenoble, France for excellent instrumental support at BM29. We also thank beamline scientists at Diamond Light Source for their help with crystallographic data collection. R.N. was supported by an MRC grant (MR/K011715/1). D.W.W. was supported by the CCP-SAS project, a joint EPSRC (EP/K039121/1) and NSF (CHE-1265821) grant. R.W. is a member of the Midland Block Allocation Group for access to Diamond Light Source (allocation grants MX8359 and MX10369).

Received: September 20, 2016
Revised: December 8, 2016
Accepted: December 21, 2016
Published: January 19, 2017

REFERENCES

Botto, M. (1998). C1q knock-out mice for the study of complement deficiency in autoimmune disease. Exp. Clin. Immunogenet. *15*, 231–234.

Carroll, M.C. (2004). The complement system in regulation of adaptive immunity. Nat. Immunol. *5*, 981–986.

Chen, C.B., and Wallis, R. (2001). Stoichiometry of complexes between mannose-binding protein and its associated serine proteases. Defining functional units for complement activation. J. Biol. Chem. *276*, 25894–25902.

Cole, J.L., Lary, J.W., P Moody, T., and Laue, T.M. (2008). Analytical ultracentrifugation: sedimentation velocity and sedimentation equilibrium. Methods Cell Biol. *84*, 143–179.

Degn, S.E., Jensen, L., Olszowski, T., Jensenius, J.C., and Thiel, S. (2013). Co-complexes of MASP-1 and MASP-2 associated with the soluble pattern-recognition molecules drive lectin pathway activation in a manner inhibitable by MAp44. J. Immunol. *191*, 1334–1345.

Degn, S.E., Kjaer, T.R., Kidmose, R.T., Jensen, L., Hansen, A.G., Tekin, M., Jensenius, J.C., Andersen, G.R., and Thiel, S. (2014). Complement activation by ligand-driven juxtaposition of discrete pattern recognition complexes. Proc. Natl. Acad. Sci. USA *111*, 13445–13450.

Dobo, J., Harmat, V., Beinrohr, L., Sebestyen, E., Zavodszky, P., and Gal, P. (2009). MASP-1, a promiscuous complement protease: structure of its catalytic region reveals the basis of its broad specificity. J. Immunol. *183*, 1207–1214.

Feinberg, H., Uitdehaag, J.C., Davies, J.M., Wallis, R., Drickamer, K., and Weis, W.I. (2003). Crystal structure of the CUB1-EGF-CUB2 region of mannose-binding protein associated serine protease-2. EMBO J. *22*, 2348–2359.

Gal, P., Harmat, V., Kocsis, A., Bian, T., Barna, L., Ambrus, G., Vegh, B., Balczer, J., Sim, R.B., Naray-Szabo, G., and Zavodszky, P. (2005). A true autoactivating enzyme. Structural insight into mannose-binding lectin-associated serine protease-2 activations. J. Biol. Chem. *280*, 33435–33444.

Gingras, A.R., Girija, U.V., Keeble, A.H., Panchal, R., Mitchell, D.A., Moody, P.C., and Wallis, R. (2011). Structural basis of mannan-binding lectin recognition by its associated serine protease MASP-1: implications for complement activation. Structure *19*, 1635–1643.

Gregory, L.A., Thielens, N.M., Arlaud, G.J., Fontecilla-Camps, J.C., and Gaboriaud, C. (2003). X-ray structure of the Ca2+-binding interaction domain of C1s. Insights into the assembly of the C1 complex of complement. J. Biol. Chem. *278*, 32157–32164.

Gregory, L.A., Thielens, N.M., Matsushita, M., Sorensen, R., Arlaud, G.J., Fontecilla-Camps, J.C., and Gaboriaud, C. (2004). The X-ray structure of human mannan-binding lectin-associated protein 19 (MAp19) and its interaction site with mannan-binding lectin and L-ficolin. J. Biol. Chem. *279*, 29391–29397.

Harmat, V., Gal, P., Kardos, J., Szilagyi, K., Ambrus, G., Vegh, B., Naray-Szabo, G., and Zavodszky, P. (2004). The structure of MBL-associated serine protease-2 reveals that identical substrate specificities of C1s and MASP-2 are realized through different sets of enzyme-substrate interactions. J. Mol. Biol. *342*, 1533–1546.

Heja, D., Kocsis, A., Dobo, J., Szilagyi, K., Szasz, R., Zavodszky, P., Pal, G., and Gal, P. (2012). Revised mechanism of complement lectin-pathway activation revealing the role of serine protease MASP-1 as the exclusive activator of MASP-2. Proc. Natl. Acad. Sci. USA *109*, 10498–10503.

Hurwitz, S. (1996). Homeostatic control of plasma calcium concentration. Crit. Rev. Biochem. Mol. Biol. *31*, 41–100.

Iwaki, D., Kanno, K., Takahashi, M., Endo, Y., Matsushita, M., and Fujita, T. (2011). The role of mannose-binding lectin-associated serine protease-3 in activation of the alternative complement pathway. J. Immunol. *187*, 3751–3758.

Jensenius, H., Klein, D.C., van Hecke, M., Oosterkamp, T.H., Schmidt, T., and Jensenius, J.C. (2009). Mannan-binding lectin: structure, oligomerization, and flexibility studied by atomic force microscopy. J. Mol. Biol. *391*, 246–259.

Kidmose, R.T., Laursen, N.S., Dobo, J., Kjaer, T.R., Sirotkina, S., Yatime, L., Sottrup-Jensen, L., Thiel, S., Gal, P., and Andersen, G.R. (2012). Structural basis for activation of the complement system by component C4 cleavage. Proc. Natl. Acad. Sci. USA *109*, 15425–15430.

Kjaer, T.R., Le le, T.M., Pedersen, J.S., Sander, B., Golas, M.M., Jensenius, J.C., Andersen, G.R., and Thiel, S. (2015). Structural insights into the initiating complex of the lectin pathway of complement activation. Structure *23*, 342–351.

Miller, A., Phillips, A., Gor, J., Wallis, R., and Perkins, S.J. (2012). Near-planar solution structures of mannose-binding lectin oligomers provide insight on the activation of the lectin pathway of complement. J. Biol. Chem. *287*, 3930–3945.

Møller-Kristensen, M., Thiel, S., Hansen, A.G., and Jensenius, J.C. (2003). On the site of C4 deposition upon complement activation via the mannan-binding lectin pathway or the classical pathway. Scand. J. Immunol. *57*, 556–561.

Ortega, A., Amoros, D., and Garcia de la Torre, J. (2011). Prediction of hydrodynamic and other solution properties of rigid proteins from atomic- and residue-level models. Biophys. J. *101*, 892–898.

Perkins, S.J., Nan, R., Li, K., Khan, S., and Abe, Y. (2011). Analytical ultracentrifugation combined with X-ray and neutron scattering: experiment and modelling. Methods *54*, 181–199.

Perkins, S.J., Wright, D.W., Zhang, H., Brookes, E.H., Chen, J., Irving, T.C., Krueger, S., Barlow, D.J., Edler, K.J., Scott, D.J., et al. (2016). Atomistic modelling of scattering data in the collaborative computational project for small angle scattering (CCP-SAS). J. App. Crystallogr. *49*, 1861–1875.

Pernot, P., Round, A., Barrett, R., De Maria Antolinos, A., Gobbo, A., Gordon, E., Huet, J., Kieffer, J., Lentini, M., Mattenet, M., et al. (2013). Upgraded ESRF BM29 beamline for SAXS on macromolecules in solution. J. Synch. Radiat. *20*, 660–664.

Porter, R.R., and Reid, K.B.M. (1978). The biochemistry of complement. Nature *275*, 699–704.

Round, A., Felisaz, F., Fodinger, L., Gobbo, A., Huet, J., Villard, C., Blanchet, C.E., Pernot, P., McSweeney, S., Roessle, M., et al. (2015). BioSAXS Sample Changer: a robotic sample changer for rapid and reliable high-throughput X-ray solution scattering experiments. Acta Crystallogr. D Biol. Crystallogr. *71*, 67–75.

Rupp, B. (2010). Biomolecular Crystallography: Principle, Practice and Application to Structural Biology (Garland Science).

Schwaeble, W.J., Lynch, N.J., Clark, J.E., Marber, M., Samani, N.J., Ali, Y.M., Dudler, T., Parent, B., Lhotta, K., Wallis, R., et al. (2011). Targeting of mannan-binding lectin-associated serine protease-2 (Masp2) confers a significant degree of protection from myocardial and gastrointestinal ischemia/reperfusion injury. Proc. Natl. Acad. Sci. USA *108*, 7523–7528.

Skjoedt, M.O., Hummelshoj, T., Palarasah, Y., Honore, C., Koch, C., Skjodt, K., and Garred, P. (2012). A novel mannose-binding lectin/ficolin-associated protein is highly expressed in heart and skeletal muscle tissues and inhibits complement activation. J. Biol. Chem. *285*, 8234–8243.

Stengaard-Pedersen, K., Thiel, S., Gadjeva, M., Moller-Kristensen, M., Sorensen, R., Jensen, L.T., Sjoholm, A.G., Fugger, L., and Jensenius, J.C. (2003). Inherited deficiency of mannan-binding lectin-associated serine protease 2. N. Engl. J. Med. *349*, 554–560.

Takahashi, M., Ishida, Y., Iwaki, D., Kanno, K., Suzuki, T., Endo, Y., Homma, Y., and Fujita, T. (2010). Essential role of mannose-binding lectin-associated serine protease-1 in activation of the complement factor D. J. Exp. Med. *207*, 29–37, S21–3.

Taylor, P.R., Carugati, A., Fadok, V.A., Cook, H.T., Andrews, M., Carroll, M.C., Savill, J.S., Henson, P.M., Botto, M., and Walport, M.J. (2000). A hierarchical role for classical pathway complement proteins in the clearance of apoptotic cells in vivo. J. Exp. Med. *192*, 359–366.

Teillet, F., Gaboriaud, C., Martin, L., Lacroix, M., Ogbi, S., Fontecilla-Camps, J.C., Arlaud, G.J., and Thielens, N.M.. (2006). The CUB1-EGF-CUB2 interaction domain of human MASP-3: X-ray structure and identification of its binding sites for MBL and L- and H-ficolins. In Proceedings of the VIth International Workshop on the First Component of Complement, C1 and Collectins. Seeheim, Germany, 15.

Teillet, F., Gaboriaud, C., Lacroix, M., Martin, L., Arlaud, G.J., and Thielens, N.M. (2008). Crystal structure of the CUB1-EGF-CUB2 domain of human MASP-1/3 and identification of its interaction sites with mannan-binding lectin and ficolins. J. Biol. Chem. *283*, 25715–25724.

Terai, I., Kobayashi, K., Matsushita, M., and Fujita, T. (1997). Human serum mannose-binding lectin (MBL)-associated serine protease-1 (MASP-1): determination of levels in body fluids and identification of two forms in serum. Clin. Exp. Immunol. *110*, 317–323.

Turner, M.W. (1996). Mannose-binding lectin: the pluripotent molecule of the innate immune system. Immunol. Today *17*, 532–540.

Venkatraman Girija, U., Gingras, A.R., Marshall, J.E., Panchal, R., Sheikh, M.A., Gal, P., Schwaeble, W.J., Mitchell, D.A., Moody, P.C.E., and Wallis, R. (2013). Structural basis of the C1q/C1s interaction and its central role in assembly of the C1 complex of complement activation. Proc. Natl. Acad. Sci. USA *110*, 13916–13920.

Wallis, R. (2007). Interactions between mannose-binding lectin and MASPs during complement activation by the lectin pathway. Immunobiology *212*, 289–299.

Wallis, R., and Drickamer, K. (1999). Molecular determinants of oligomer formation and complement fixation in mannose-binding proteins. J. Biol. Chem. *274*, 3580–3589.

Wallis, R., and Dodd, R.B. (2000). Interaction of mannose-binding protein with associated serine proteases: effects of naturally occurring mutations. J. Biol. Chem. *275*, 30962–30969.

Wallis, R., Shaw, J.M., Uitdehaag, J., Chen, C.B., Torgersen, D., and Drickamer, K. (2004). Localization of the serine protease-binding sites in the collagen-like domain of mannose-binding protein: indirect effects of naturally occurring mutations on protease binding and activation. J. Biol. Chem. *279*, 14065–14073.

Wallis, R., Dodds, A.W., Mitchell, D.A., Sim, R.B., Reid, K.B., and Schwaeble, W.J. (2007). Molecular interactions between MASP-2, C4, and C2 and their activation fragments leading to complement activation via the lectin pathway. J. Biol. Chem. *282*, 7844–7851.

Wallis, R., Mitchell, D.A., Schmid, R., Schwaeble, W.J., and Keeble, A.H. (2010). Paths reunited: initiation of the classical and lectin pathways of complement activation. Immunobiology *215*, 1–11.

Wright, D.W., and Perkins, S.J. (2015). SCT: a suite of programs for comparing atomistic models with small-angle scattering data. J. App. Crystallogr. *48*, 953–961.

Structure, Volume *25*

Supplemental Information

Flexibility in Mannan-Binding Lectin-Associated Serine Proteases-1 and -2 Provides Insight on Lectin Pathway Activation

Ruodan Nan (南若丹), Christopher M. Furze, David W. Wright, Jayesh Gor, Russell Wallis, and Stephen J. Perkins

SUPPLEMENTARY INFORMATION

FLEXIBILITY IN MANNAN-BINDING LECTIN-ASSOCIATED SERINE PROTEASES-1 AND -2 PROVIDES NOVEL INSIGHT ON LECTIN PATHWAY ACTIVATION

Ruodan Nan (南若丹)[1], Christopher M. Furze[2], David W. Wright[1], Jayesh Gor[1], Russell Wallis[2], and Stephen J. Perkins[1]

From the [1]Department of Structural and Molecular Biology, Division of Biosciences, Darwin Building, University College London, Gower Street, London WC1E 6BT, U.K.; [2]Departments of Infection, Immunity and Inflammation and Molecular Cell Biology, Maurice Shock Medical Sciences Building, University of Leicester, University Road, Leicester, LE1 9HN, U.K.

***Contact**: Prof S. J. Perkins, Department of Structural and Molecular Biology, Darwin Building, University College London, Gower Street, London, WC1E 6BT, U.K. Tel: 020-7679-7048, FAX: 020-7679-7193; Email: s.perkins@ucl.ac.uk.

Table S1, related to Figure 8 and Figure S2. The mean values for the distances *D1*, *D2* and *D3* in the six MASP proteins, and the angles *θ1*, *θ2* and *θ3* for the starting structures and the best-fit models.

	Starting structures *				Ten best-fit models *			
	Distance (nm)		Angle (°)		Distance (nm)		Angle (°)	
	Mean *D1* and *D2*	*D3*	*θ3*	*θ1* and *θ2*	Mean *D1* and *D2*	*D3*	*θ3*	*θ1* and *θ2*
MASP-1 3D	5.0	10.0	171	4.5	5.1 ± 0.2	9.5 ± 0.5	142 ± 8	19 ± 4
MASP-2 3D	4.9	9.8	168	6.0	4.9 ± 0.2	9.5 ± 0.3	156 ± 9	12 ± 4
Zymogen MASP-1	14.3	28.6	172	3.9	12.0 ± 0.6	20.3 ± 0.9	117 ± 7	32 ± 3
Activated MASP-1	13.3	26.5	168	5.9	11.9 ± 1.2	22.4 ± 1.1	142 ± 13	19 ± 7
Zymogen MASP-2	12.4	24.7	166	7.1	10.8 ± 2.5	16.2 ± 2.1	100 ± 29	40 ± 19
Activated MASP-2	12.3	24.4	166	7.0	11.2 ± 1.7	20.1 ± 1.3	127 ± 10	26 ± 7

* The distances *D1*, *D2* and *D3* and the angles *θ1*, *θ2* and *θ3* for the starting structures and the best-fit models are defined in Figure S2. The values for full-length MASP-1 and MASP-2 are from Search 1.

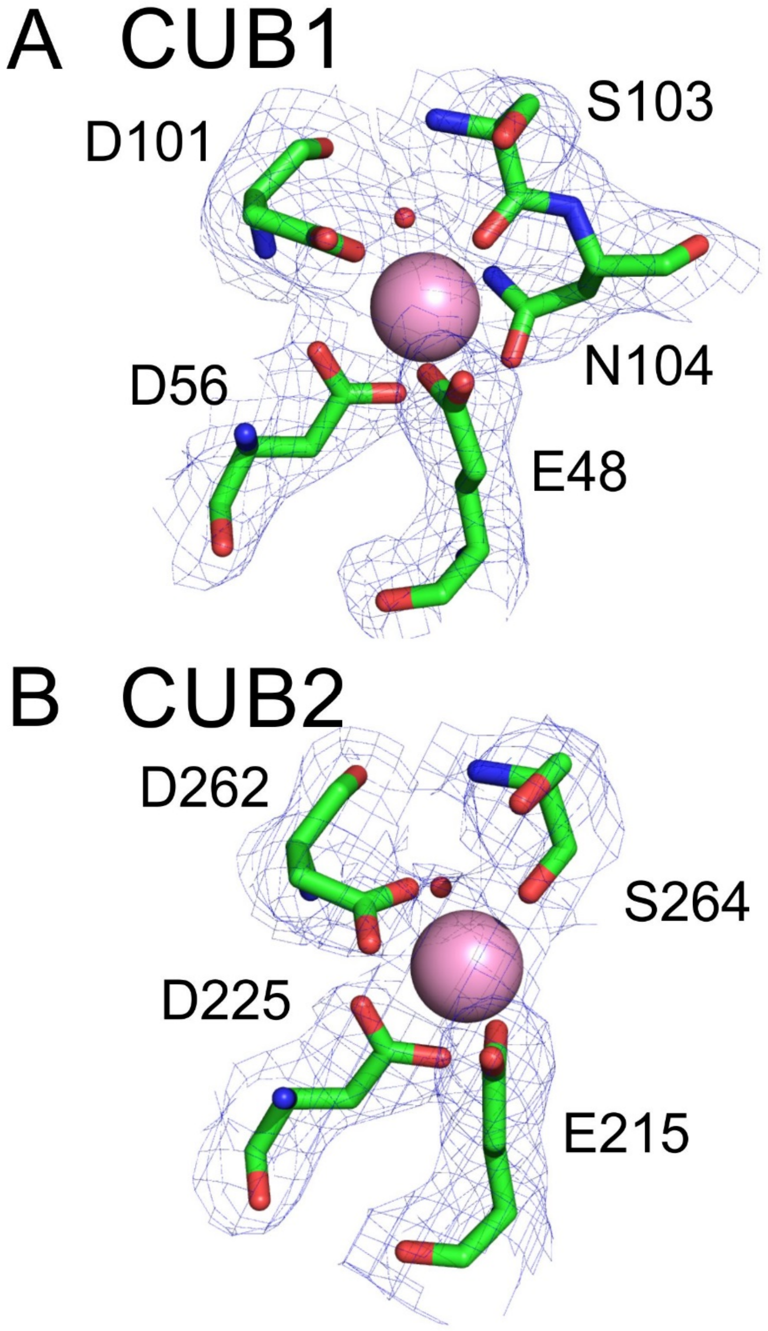

Figure S1, related to Figure 3B. Structure of the calcium binding sites in the CUB1 (A) and CUB2 (B) domains of MASP-2 from the structure PDB code 5CIS. The Ca^{2+} atom is shown as a pink sphere; a single water molecule is shown as a small red sphere. The electron density map is shown by the blue mesh (σ = 1).

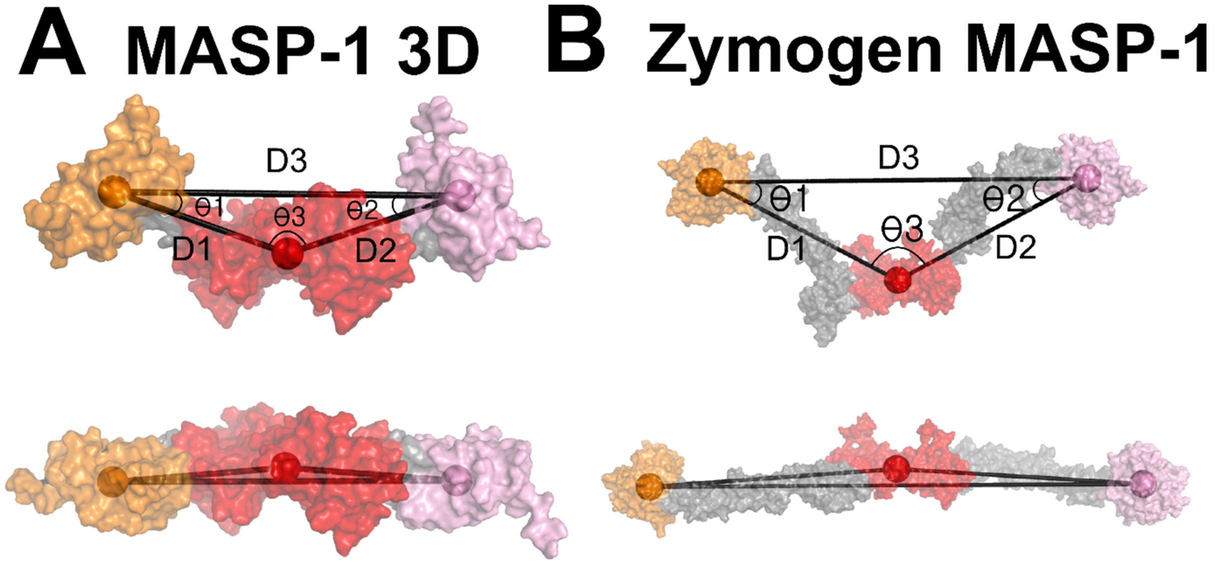

Figure S2, related to Figure 8 and Table S1. Comparison of the starting structure and best-fit models for MASP 3D and MASP. The distances D1-D3 and angles θ1-θ3 between the central and end regions of the MASP models defined their overall shapes (Table S1). *A*, The side-on view of the best-fit structure of MASP-1 3D (upper) is representative of MASP-1 3D and MASP-2 3D. This is compared in the same view with its crystal structure (lower). Its centre of mass is defined as the centre of the four CUB1-EGF domains (red). Two more centres of mass are defined as the CUB2 domain of monomer 1 (Arm 1; orange), and the CUB2 domain of monomer 2 (Arm 2; pink). *B*, The side-on view of the best-fit zymogen MASP-1 structure (upper) is representative of full-length MASP-1 and MASP-2. This is compared in the same view with its starting homology model used to initiate the scattering fits (lower). Its centre of mass is the centre of the four CUB1-EGF domains (red). Two more centres of mass are defined in the serine protease domains of monomer 1 (orange) and monomer 2 (pink).

EXPERIMENTAL PROCEDURES

Production, purification and compositions of the MASP proteins

In this study, all sequences were numbered according to the first residue in the mature protein, and not from the start of the signal peptide in accordance with previous structural analysis of MASPs. The rat zymogen MASP-1 was produced in Chinese hamster ovary cells by substituting the five residues immediately preceding the cleavage site for MASP-1 activation 425-KHISR-429 with DDDDK, where the latter is the recognition sequence for the serine protease enterokinase (Figure 1) (Chen and Wallis, 2001, 2004). This substitution prevented MASP-1 auto-activation. The rat zymogen MASP-2 form was produced by mutating Ser613 at its catalytic active site to Ala613 (Figure 1). Both zymogen MASP-1 and MASP-2 were synthesised with N-terminal hexahistidine tags and purified by nickel affinity chromatography, followed by size-exclusion gel filtration to remove aggregates and minor contaminants (Chen and Wallis, 2001, 2004). MASP-1 was activated by incubating the zymogen MASP-1 with enterokinase at a ratio of 0.01% w/w at 37°C in 20 mM Tris, 140 mM NaCl, 2 mM $CaCl_2$, pH 7.4 in a water bath for 4 h. The reaction was stopped by adding 1% w/w soybean trypsin inhibitor. MASP-2 was activated by incubating the zymogen MASP-2 at 1 mg/ml mixed with bovine trypsin at 10 µg/ml in 20 mM Tris, 140 mM NaCl, 0.1 mM EDTA, pH 7.4, for 210 s at 37°C in a water bath. The reaction was stopped by adding 20 µg/ml soybean trypsin inhibitor to the mixture and placing the reaction mixture immediately on ice. All proteins were purified by gel filtration chromatography on a Superdex 200 16/60 column (GE Healthcare), prior to crystallisation or further analysis. SDS-PAGE was used to check the purity of all the samples.

Zymogen MASP-1 and MASP-2 migrated as single band at about 80 kDa by SDS-PAGE under both reducing and non-reducing conditions. On activation, MASP-1 and MASP-2 showed a single band at 80 kDa in non-reducing conditions, and two bands at 30 kDa and 50 kDa in reducing conditions that correspond to the cleavage site just before the SP domain, which is disulphide linked but separate from the other five MASP domains. The MASP-1 3D and MASP-2 3D fragments showed two closely-positioned bands at 40 kDa, which were attributed to alternatively-glycosylated forms. Thus the major bands for the MASP monomers observed by SDS-PAGE confirm the purifications of disulphide-linked zymogen and activated MASP as previously described (Chen and Wallis, 2001; Wallis and Dodd, 2000). The absorbance coefficients (1%, 280 nm, 1 cm path length) for MASP-1 3D, zymogen MASP-1, activated MASP-1, MASP-2 3D, zymogen MASP-2 and activated MASP-2 were calculated to be 12.1, 14.5, 14.5, 12.3, 14.8 and 14.8 respectively. Molecular masses were calculated from the sequences to be 74.9 kDa for MASP-1 3D, 169.9 kDa for zymogen and activated MASP-1, 73.7 kDa for MASP-2 3D, and 162.7 kDa for zymogen and activated MASP-2, all in their dimeric forms. These masses assumed that one N-linked biantennary type oligosaccharide was present at each of the Asn40, Asn169 and Asn398 sites in zymogen and activated MASP-1, and the Asn94, Asn276 and Asn632 sites in zymogen and activated MASP-2 (Figure 1).

MASP crystallization, data collection and analysis

Proteins in 20 mM Tris pH 7.5 containing 20 mM NaCl and 2 mM $CaCl_2$ were crystallised using the sitting-drop vapour diffusion method by mixing equal volumes of protein (4 - 10 mg/mL) and crystallisation buffer at 20°C. The MASP-2 3D fragment was crystallised in three different conditions: 100 mM Tris pH 8.0 containing 40% 2-methyl-2,4-pentanediol and 80 mM ammonium acetate; 100 mM Tris pH 8.0 containing 40% 2-methyl-2,4-pentanediol; and 200 mM sodium citrate and 100 mM Tris pH 8.5 containing 80 mM ammonium acetate and 20% polyethylene glycol 20K. The MASP-1 3D fragment was crystallised in 100 mM imidazole/2-(*N*-morpholino)ethanesulphonic acid buffer at pH 6.5, containing 20% ethylene glycol and 10% polyethylene glycol 8K together with a mixture of 30 mM diethylene glycol, 30 mM triethylene glycol, 30 mM tetraethylene glycol and 30 mM pentaethylene glycol. Crystals were stored in liquid nitrogen and were maintained at 100 K during data collection. Diffraction data were collected at Leicester on a rotating copper anode home X-ray source and at the Diamond Light Source, and were processed with XDS (Kabsch, 1993) or Mosflm (Battye et al., 2011). Phases were determined by molecular replacement with Phaser (McCoy et al., 2007) using the crystal structures of human MASP-1 (PDB code: 3DEM) and rat MASP-2 (PDB code: 1NTO) as search models. Models were optimized by cycles of manual refinement with Coot (Emsley and Cowtan, 2004) and

refinement in Refmac5, part of the CCP4 software suite (Collaborative Computational Project, 1994), and in Phenix (Adams et al., 2010). The final refined crystal structures were deposited into the Protein Data Bank with accession codes 5CKN (MASP-2 3D in acetate), 5CIS (MASP-2 3D in 20% polyethylene glycol), and 5CKM (MASP-2 3D in citrate) and 5CKQ (MASP-1 3D), in that order. Calculations of buried surface areas at the dimer interface were made from PISA analyses (Krissinel and Henrick, 2007).

For MASP-1, the 12 contact residues (i.e. showing more than 50% buried accessible surface areas) at the dimer interface were Phe10, Met44, His115, Met117, Ala118, His139, Asn140, Tyr141, Ile142, Tyr146, Ser148 and Phe151. These 12 contact residues comprised 79% of the total buried accessible surface area. For the three MASP-2 structures, these 15-18 dimer interface contact residues were determined to be (Pro6), Phe8, Phe36, Arg39, Tyr41, Thr43, Glu112, Phe114, Ala116, Ala117, (Tyr136), His138, Asn139, Tyr140, Leu141, (Tyr145), Ser147 and Val150. These 15-18 dimer contact residues comprised 80-89% of the total buried accessible surface area. The majority of these contacts were hydrophobic in character. Two hydrophilic exceptions in MASP-2 included Arg39 that formed a hydrogen bond with a C=O of Asn139 across the dimer interface, and Glu110 that made a salt bridge with Arg10 within the same MASP molecule. All these contact residues were present in the human MASP-1 and MASP-2 sequences except for Phe36, Tyr136, Tyr140 and Val150 in rat MASP-2, which were replaced by the similar Tyr, His, Tyr and Ala residues in human MASP-2, respectively. This provides further evidence that a well-defined MASP dimer was physiologically important in both the human and rat proteins.

X-ray scattering data for MASP

X-ray scattering curves *I(Q)* were acquired in two beam sessions on the BioSAXS robot at Instrument BM29 at the European Synchrotron Radiation Facility, Grenoble, France (Pernot et al., 2013; Round et al., 2015). Data were recorded using a CMOS hybrid pixel Pilatus 1M detector with a resolution of 981 x 1043 pixels (pixel size of 172 µm × 172 µm). One session was operated with a ring energy of 6.0 GeV in 7/8 + 1 multibunch mode, and the other session was operated with a ring energy of 6.0 GeV in 24*8 + 1 filling mode. The sample-detector distance was 2.867 m, the X-ray wavelength was 0.09919 nm, and the diameter of the flow cell quartz capillary was 1.8 mm in both sessions. Potential radiation damage was eliminated by the continuous movement of the sample in the flow cell during beam exposure, the use of 10 time frames with an exposure time of 1 s per frame, and on-line checks for the absence of radiation damage. The scattering data were collected for MASP-1 3D at 0.31-0.78 mg/ml, zymogen MASP-1 at 0.25-1.26 mg/ml, and activated MASP-1 at 0.26-0.43 mg/ml in Tris buffer with 2 mM Ca^{2+}. Data were collected for MASP-2 3D at 0.30-0.60 mg/ml, zymogen MASP-2 at 0.30-0.68 mg/ml, and activated MASP-2 at 0.29 mg/ml in Tris buffer with EDTA. The MASP-2 samples were also measured with Ca^{2+} present, when 4 mM $CaCl_2$ was added immediately before measurements for MASP-2 3D at 0.30-0.60 mg/ml, zymogen MASP-2 at 0.3-0.5 mg/ml, and activated MASP-2 at 0.29 mg/ml. Data reduction was performed using ISpyB software (De Maria Antolinos et al., 2015).

In a given solute-solvent contrast, the radius of gyration R_g corresponds to the mean square distance of scattering elements from their centre of gravity, and is a measure of structural elongation. Guinier analyses of the *I(Q)* curves at low scattering vectors Q (where $Q = 4\pi \sin \theta/\lambda$; 2θ is the scattering angle and λ is the wavelength) give the R_g value and the forward scattering at zero angle $I(0)$ from the expression (Glatter and Kratky, 1982):

$$\ln I(Q) = \ln I(0) - R_g^2\, Q^2/3$$

This expression is valid in a $Q.R_g$ range up to 1.5. Linear R_g plots were obtained in Q ranges of 0.23-0.38 nm^{-1} for both MASP 3Ds and 0.08-0.175 nm^{-1} for the full-length MASPs. If the structure is elongated, For elongated structures, the cross-sectional radius of gyration R_{xs} and cross-sectional intensity at zero angle $[I(Q)Q]_{Q\to 0}$ is obtained from analyses at larger Q ranges:

$$\ln [I(Q)Q] = [I(Q)Q]_{Q\to 0} - R_{xs}^2 Q^2/2$$

For MASP 3D, the linear R_{xs} region corresponded to a Q range of 0.45-0.75 nm^{-1}. For the full-length zymogen and activated MASP, the four $I(Q).Q$ vs Q^2 plots showed linear R_{xs} regions in the Q range of

0.60-0.86 nm^{-1}. The Guinier analyses were performed using SCT software (Miller et al., 2012; Wright and Perkins, 2015).

Indirect transformation of the $I(Q)$ curve measured in reciprocal space into real space gives the distance distribution function $P(r)$ and was carried out using the program GNOM (Semenyuk and Svergun, 1991):

$$P(r) = \frac{1}{2\pi^2} \int_o^\infty I(Q)\ Qr\ \sin(Qr)\ dQ$$

$P(r)$ corresponds to the distribution of distances r between volume elements, and gives an alternative calculation of the R_G and $I(0)$ values based on the full scattering curve $I(Q)$ following an assumption of the maximum dimension (D_{max}). This provided structural information in real space. It also gives the most frequently occurring distance M and the maximum dimension of the macromolecule L. For the MASP proteins, the X-ray curves utilised up to 497 data points in the Q range from 0.06 nm^{-1} to 2.20 nm^{-1} for MASP-1 3D and MASP-2 3D, from 0.14 nm^{-1} to 2.20 nm^{-1} for zymogen and activated MASP-1 and MASP-2. Other details are described elsewhere (Fernando et al., 2007; Nan et al., 2008; Okemefuna et al., 2008).

Sedimentation velocity data for MASP

Analytical ultracentrifugation data were obtained on Beckman XL-I instruments equipped with AnTi50 rotors and using two-sector cells with column heights of 12 mm at a rotor speed of 50,000 rpm. Sedimentation velocity experiments were performed at 20 °C for MASP-1 3D at 0.71 mg/ml, zymogen MASP-1 at 0.28 mg/ml, activated MASP-1 at 0.34 mg/ml (all three in 20 mM Tris, 140 mM NaCl, 2 mM $CaCl_2$, pH 7.4), and for MASP-2 3D at 0.32 mg/ml, zymogen MASP-2 at 0.14 mg/ml, and activated MASP-2 at 0.14 mg/ml (all three in a Tris buffer with EDTA, namely 20 mM Tris, 140 mM NaCl, 0.1 mM EDTA, pH 7.4). Sedimentation was monitored using absorbance optics at 280 nm. The sedimentation coefficient distribution $c(s)$ analyses were performed by fitting up to 60 absorbance scans directly to the Lamm equation using SEDFIT software version 14.6e (Schuck, 1998, 2000). In the $c(s)$ analyses, the meniscus, the bottom of the cell, the baseline, and the average frictional ratio f/f_0 were floated until the overall root mean deviation and the fits between the observed and calculated sedimentation boundaries were satisfactory. Here, f is the frictional coefficient of the macromolecule and f_0 is the frictional coefficient of the sphere with the same hydrated volume as the macromolecule. The starting f/f_0 values were 1.50 for MASP-1 3D and MASP-2 3D, and 1.85 for zymogen and activated MASP-1 and MASP-2. The partial specific volume v values were as follows: MASP-1 3D, 0.709 ml/g; zymogen MASP-1, 0.721 ml/g; activated MASP-1, 0.721 ml/g; MASP-2 3D, 0.715 ml/g; zymogen MASP-2, 0.725 ml/g; and activated MASP-2, 0.725 ml/g (Perkins, 1986). The buffer densities were measured using an Anton Paar DMA 5000 density meter, and compared with the theoretical values calculated by SEDNTERP (Laue et al., 1992). This resulted in a density of 1.00576 g/ml for Tris-Ca buffer at 20°C, compared to a theoretical value of 1.00576 g/ml, and one of 1.00559 g/ml for Tris-EDTA at 20°C.

Generation of starting structural models for MASP

Atomistic scattering modelling fits for each of the six proteins was performed in order to define their solution structures. For each protein, a starting model was constructed as follows, after which each model was subjected to two sets of conformational randomisation to generate broad ranges of trial structures for assessment. For each protein, the first search (Search 1) was initiated directly from the starting model detailed below. The second search (Search 2), was performed as a control of reproducibility and was seeded with the best fit scattering models from Search 1:

(i,ii) Rat MASP-1 3D and rat MASP-2 3D: For these initial $s_{20,w}$ calculations and scattering curve fits, the starting structures were the crystal structures for rat MASP-1 3D and rat MASP-2 3D bound with Ca^{2+} from this study (Figure 2).

(iii) Rat zymogen MASP-1 dimer: For the initial fits, the starting structure was based on three experimental structures: (a) the crystal structure for rat MASP-1 3D with Ca^{2+} (Figure 2*A*), (b) the

crystal structure for human mannose-binding lectin (MBL)/ficolin-associated protein-1 (MAP-1) which is the result of a differential splicing of MASP-1 gene and includes the CUB1-EGF-CUB2-SCR1 fragment of MASP-1 (PDB code: 4AQB) (Skjoedt et al., 2012a), and (c) the crystal structure of the SCR1-SCR2-SP fragment of human MASP-1 proenzyme (PDB code: 3GOV) (Dobo et al., 2009). Using MODELLER v9.11 (Sali and Blundell, 1993), a homology model for the rat MASP-1 CUB2-SCR1 fragment was constructed from the CUB2-SCR1 fragment of human MAP-1 (PDB code: 4AQB) as template. The homology model for the rat zymogen MASP-1 SCR1-SCR2-SP fragment was constructed from this fragment in the human MASP-1 crystal structure (PDB code: 3GOV). The homology model for the full-length rat zymogen MASP-1 monomer was formed using multi-templates to make the full monomer from structures for each of the three CUB1-EGF-CUB2, CUB2-SCR2 and SCR1-SCR2-SP fragments. The resulting monomer structure was superimposed onto the CUB1-EGF dimer in the crystal structure of MASP-1 3D dimer in order to form the full-length dimer (Figure 2*A*).

(iv) Activated MASP-1 dimer: The starting dimer structure was constructed using the same strategy for the zymogen, but replacing the template SCR1-SCR2-SP structure with the crystal structure of the SCR1-SCR2-SP fragment in catalytically active human MASP-1 (PDB code: 4DJZ) (Heja et al., 2012).

(v) Zymogen MASP-2 dimer: The starting structure was constructed in the same way as for MASP-1. The full-length rat zymogen MASP-2 structure was formed using the crystal structure for the rat MASP-2 3D dimer with Ca^{2+} (Figure 2*B*), combined with two rat homology models for the CUB2-SCR1 fragment based on the crystal structure of human MAP-1 (PDB code: 4AQB) and the SCR1-SCR2-SP fragment based on the crystal structure for human MASP-2 in its proenzyme form (PDB code: 1ZJK) (Gal et al., 2005).

(vi) Activated MASP-2 dimer: The starting structure for the homology model was based on the crystal structure of rat MASP-2 3D (Figure 2*B*). This was supplemented with three homology models constructed from the CUB2-SCR1 fragment in the crystal structure for human MAP-1 (PDB code: 4AQB), the SCR1-SCR2-SP fragment in the crystal structure for the human MASP-2 proenzyme (PDB code: 1ZJK) (Gal et al., 2005), and the crystal structure of the catalytically active human MASP-2 SCR2-SP fragment (PDB code: 3TVJ) (Heja et al., 2012).

Scattering curve calculation using SCT

Scattering curve simulations involved the generation of up to 30,000 conformationally-randomised domain arrangements of the six MASP proteins, followed by scattering curve calculations. The calculated curves were compared with the experimental X-ray scattering curves in order to identify the best fit solution structures (Perkins et al., 2009; Perkins et al., 2011; Perkins et al., 2016). For this, hydrogen atoms were added to all the starting MASP structures, each of which was parameterized using the CHARMM36 forcefield (Guvench et al., 2009; Hatcher et al., 2009; MacKerell et al., 1998; Mackerell et al., 2004; Raman et al., 2010) via the CHARMM-GUI website (http://www.charmm-gui.org/) (Jo et al., 2008; Jo et al., 2011) in order to generate parameters for the following structure simulations. Likely regions of linker flexibility between the six MASP domains and the His-tag in the starting structures were identified in bold (Figure 1). For the rat MASP-1 3D dimer, the flexible linkers were set as 162-RVE-164, and 277-HHHHHH-282. For rat zymogen and activated MASP-1, the flexible linkers were 1-HHHHHH-6, 173-RVE-175, 288-AGNE-291, 354-KIVD-357, and 424-LPV-426. For the rat MASP-2 3D dimer, the flexible linkers were 163-AL-164, and 279-HHHHHH-284. For rat zymogen and activated MASP-2, the flexible linkers were 1-HHHHHH-6, 173-AL-174, 287-TAQP-290, 353-SIID-356 and 421-KPV-423. The flexible linker conformations were randomly sampled using the dihedral angle Monte Carlo module of the SASSIE package (Curtis et al., 2012). The backbone φ and ψ angles of the peptide bonds within these linkers were varied independently of one another. The maximum rotation angle steps for both φ and ψ angles were 80°. Because the linkers and domains were considered together when creating each trial MASP structure, steric overlaps were handled better, in which sterically-poor models were discarded by SASSIE at their creation, and not later as in the classic SCT package (Miller et al., 2012). The Monte Carlo simulations generated 4,517 trial atomistic models for MASP-1 3D, 7,662 and 8,358 models for Search 1 and 2 respectively for zymogen MASP-1, 30,910 and 8,685 models for Search 1 and Search 2 for activated MASP-1, 1,982 models for MASP-2 3D, 7,662 and 8,685 models for Search 1 and Search 2 for zymogen MASP-2, and 6,173 and 8,685 models from Search 1 and Search 2 for activated MASP-2.

Each set of models generated by the Monte Carlo simulations was submitted to the SCT software suite to calculate scattering curves for comparison to the experimental X-ray scattering data (Wright and Perkins, 2015). The models were coarse-grained into small sphere models using a grid with cube-side length of 0.530 nm and a cut-off of four atoms. These parameters were optimized by SCT to produce unhydrated protein models with the correct volume calculated from the sequence. For X-ray scattering curve calculations, a hydration sphere shell corresponding to 0.3 g of water per gram of glycoprotein was added to each unhydrated model (Perkins, 1986, 2001; Wright and Perkins, 2015). Scattering curves *I(Q)* were calculated from the hydrated sphere models using the Debye equation adapted to spheres (Wright and Perkins, 2015). The calculated scattering curves from the sphere models were then compared to the experimental scattering data using a goodness of fit *R*-factor in a *Q* range extending to 2.2 nm^{-1}. The Guinier R_g and R_{xs} values were calculated from the *I(Q)* curves from the sphere models using the same *Q* ranges used to determine the experimental R_g and R_{xs} values. In each fit analysis, the best ten models were selected using three filters, namely the R_g, R_{xs}, and *R*-factor values.

To complete each search fit, one N-linked biantennary type oligosaccharide was added to each of Asn-40, Asn-169 and Asn-398 for each best-fit model of zymogen and activated MASP-1. The core oligosaccharide structure was taken from the crystal structure of the Fc fragment of human IgG1 to which additional terminal residues were added (Boehm et al., 1999). For each best-fit model of zymogen and activated MASP-2, oligosaccharide was added to each of Asn-94, Asn-276 and Asn-632. These best-fit models with carbohydrate added were resubmitted to SCT to calculate the final R_G, R_{XS} and *R*-factor values.

Utility of atomistic scattering modelling

Atomistic scattering modelling offers unique views of the multidomain complement protein structures in solution, which reinforces the interpretation of crystal structures and provides important new insights (Rodriguez et al., 2015). This method has a good track record with 77 structure determinations (Wright and Perkins, 2015), as exemplified by four examples: (i) Our scattering modelling of the SCR-6/8 domains of complement factor H predated its crystal structure and both methods revealed the same inter-SCR domain arrangement (Fernando et al., 2007; Prosser et al., 2007); (ii) The J-shaped scattering structure of factor H SCR-1/5 was used to solve the crystal structure of its complex with C3b (Okemefuna et al., 2008; Wu et al., 2009); (iii) The J-shaped structure of the five-domain SCR-1/5 rat CrrY protein was confirmed by its subsequent crystal structure (Aslam et al., 2003; Roversi et al., 2011a); (iv) The scattering modelling of a compact five-domain structure for complement factor I was confirmed by its crystal structure 13 years later (Chamberlain et al., 1998; Roversi et al., 2011b).

For the MASP proteins, crystal structures for four N-terminal CUB1-EGF-CUB2 and six C-terminal SCR1-SCR2-SP fragments were previously available, together with one more showing how the CUB2-SCR1 domains were connected (Dobo et al., 2009; Feinberg et al., 2003; Gal et al., 2005; Gingras et al., 2011; Gregory et al., 2003; Harmat et al., 2004; Heja et al., 2012; Kidmose et al., 2012; Skjoedt et al. 2012; Teillet et al., 2008). The similarity of the domain arrangements in these structures gave rise to the expectation that MASP only has one single conformation. Here, the utility of atomistic modelling was two-fold. First, in MASP 3D, atomistic modelling revealed that the EGF-CUB2 junction exhibited varied structures in solution, implying considerable flexibility. Second, in the full-length MASP proteins, atomistic modelling indicated flexibility at potentially all four linkers (EGF-CUB2, CUB2-SCR1, SCR1-SCR2 and SCR2-SP), although it was not possible to identify the degree of flexibility at each linker. This flexibility, together with the glycosylation of MASP-1 and MASP2, may explain why the full-length MASP proteins have not been crystallised so far.

The present atomistic modelling of MASP benefitted from a new methodology SASSIE to generate physically-realistic and conformationally-randomised domain arrangements based on molecular dynamics and Monte Carlo simulations (Curtis et al., 2012; Wright and Perkins, 2015; Perkins et al., 2016). Earlier crystallography and scattering analyses revealed that human and mouse IgG antibodies were not necessarily two-fold symmetric (Harris et al., 1998; Rayner et al., 2014, 2015;

Saphire et al., 2001). Thus two-fold symmetry was not assumed for the MASP dimer modelling. During each SASSIE model generation, typically 30,000 randomised linker conformations in MASP-1 and MASP-2 were generated by Monte Carlo variations of 20 and 19 θ and ψ angles. Our earlier methodology produced 10-fold less models. Such a Monte Carlo approach is rapid; 50,000 structures were sampled in less than an hour on a single CPU. Such a single-search approach is simple and does not require a range of assumptions to create molecular models (Kjaer et al., 2015). Because the MASP domains were simultaneously considered together in each trial MASP structure, sterically-poor models showing overlaps were discarded at their creation, rather than later. The availability of a large number of randomised models improved the quality of the resulting curve fits in a Q range out to 1.5 nm^{-1} with an R factor as low as 4.1% (Figure 7). The present MASP analyses indicate that atomistic modelling will continue to be useful in structure-function studies of multidomain proteins.

REFERENCES

Adams, P.D., Afonine, P.V., Bunkoczi, G., Chen, V.B., Davis, I.W., Echols, N., Headd, J.J., Hung, L.W., Kapral, G.J., Grosse-Kunstleve, R.W., *et al.* (2010). PHENIX: a comprehensive Python-based system for macromolecular structure solution. Acta Cryst. D*66*, 213-221.

Aslam, M., Guthridge, J.M., Hack, B.K., Quigg, R.J., Holers, V.M., and Perkins, S.J. (2003). The extended multidomain solution structures of the complement protein Crry and its chimeric conjugate Crry-Ig by scattering, analytical ultracentrifugation and constrained modelling: implications for function and therapy. J. Mol. Biol. *329*, 525-550.

Battye, T.G., Kontogiannis, L., Johnson, O., Powell, H.R., and Leslie, A.G. (2011). iMOSFLM: a new graphical interface for diffraction-image processing with MOSFLM. Acta Cryst. D*67*, 271-281.

Boehm, M.K., Woof, J.M., Kerr, M.A., and Perkins, S.J. (1999). The Fab and Fc fragments of IgA1 exhibit a different arrangement from that in IgG: a study by X-ray and neutron solution scattering and homology modelling. J. Mol. Biol. *286*, 1421-1447.

Chamberlain, D., Ullman, C.G., and Perkins, S.J. (1998). Possible arrangement of the five domains in human complement factor I as determined by a combination of X-ray and neutron scattering and homology modeling. Biochemistry *37*, 13918-13929.

Chen, C.B., and Wallis, R. (2001). Stoichiometry of complexes between mannose-binding protein and its associated serine proteases. Defining functional units for complement activation. J. Biol. Chem. *276*, 25894-25902.

Chen, C.B., and Wallis, R. (2004). Two mechanisms for mannose-binding protein modulation of the activity of its associated serine proteases. J. Biol. Chem. *279*, 26058-26065.

Collaborative Computational Project, N. (1994). The CCP4 Suite: Programs for Protein Crystallography. Acta. Cryst. *D50*, 760-763.

Curtis, J.E., Raghunandan, S., Nanda, H., and Krueger, S. (2012). SASSIE: A program to study intrinsically disordered biological molecules and macromolecular ensembles using experimental scattering restraints. Comput. Phys. Commun *183*, 382-389.

De Maria Antolinos, A., Pernot, P., Brennich, M.E., Kieffer, J., Bowler, M.W., Delageniere, S., Ohlsson, S., Malbet Monaco, S., Ashton, A., Franke, D., *et al.* (2015). ISPyB for BioSAXS, the gateway to user autonomy in solution scattering experiments. Acta Cryst. D*71*, 76-85.

Dobo, J., Harmat, V., Beinrohr, L., Sebestyen, E., Zavodszky, P., and Gal, P. (2009). MASP-1, a promiscuous complement protease: structure of its catalytic region reveals the basis of its broad specificity. J. Immunol. *183*, 1207-1214.

Emsley, P., and Cowtan, K. (2004). Coot: model-building tools for molecular graphics. Acta Cryst. D*60*, 2126-2132.

Feinberg, H., Uitdehaag, J.C., Davies, J.M., Wallis, R., Drickamer, K., and Weis, W.I. (2003). Crystal structure of the CUB1-EGF-CUB2 region of mannose-binding protein associated serine protease-2. EMBO J. *22*, 2348-2359.

Fernando, A.N., Furtado, P.B., Clark, S.J., Gilbert, H.E., Day, A.J., Sim, R.B., and Perkins, S.J. (2007). Associative and structural properties of the region of complement factor H encompassing the Tyr402His disease-related polymorphism and its interactions with heparin. J. Mol. Biol. *368*, 564-581.

Gal, P., Harmat, V., Kocsis, A., Bian, T., Barna, L., Ambrus, G., Vegh, B., Balczer, J., Sim, R.B., Naray-Szabo, G., and Zavodszky, P. (2005). A true autoactivating enzyme. Structural insight into mannose-binding lectin-associated serine protease-2 activations. J. Biol. Chem. *280*, 33435-33444.

Gingras, A.R., Girija, U.V., Keeble, A.H., Panchal, R., Mitchell, D.A., Moody, P.C., and Wallis, R. (2011). Structural basis of mannan-binding lectin recognition by its associated serine protease MASP-1: implications for complement activation. Structure *19*, 1635-1643.

Glatter, O., and Kratky, O. (1982). Small angle X-ray scattering (New York: Academic Press).

Gregory, L.A., Thielens, N.M., Arlaud, G.J., Fontecilla-Camps, J.C., and Gaboriaud, C. (2003). X-ray structure of the Ca2+-binding interaction domain of C1s. Insights into the assembly of the C1 complex of complement. J. Biol. Chem. *278*, 32157-32164.

Guvench, O., Hatcher, E.R., Venable, R.M., Pastor, R.W., and Mackerell, A.D. (2009). CHARMM Additive All-Atom Force Field for Glycosidic Linkages between Hexopyranoses. J. Chem. Theory Comput. *5*, 2353-2370.

Harmat, V., Gal, P., Kardos, J., Szilagyi, K., Ambrus, G., Vegh, B., Naray-Szabo, G., and Zavodszky, P. (2004). The structure of MBL-associated serine protease-2 reveals that identical substrate specificities of C1s and MASP-2 are realized through different sets of enzyme-substrate interactions. J. Mol. Biol. *342*, 1533-1546.

Harris, L.J., Larson, S.B., Skaletsky, E., and McPherson, A. (1998). Comparison of the conformations of two intact monoclonal antibodies with hinges. Immunol. Rev. *163*, 35-43.

Hatcher, E., Guvench, O., and Mackerell, A.D., Jr. (2009). CHARMM Additive All-Atom Force Field for Acyclic Polyalcohols, Acyclic Carbohydrates and Inositol. J. Chem. Theory Computat. *5*, 1315-1327.

Heja, D., Kocsis, A., Dobo, J., Szilagyi, K., Szasz, R., Zavodszky, P., Pal, G., and Gal, P. (2012). Revised mechanism of complement lectin-pathway activation revealing the role of serine protease MASP-1 as the exclusive activator of MASP-2. Proc. Natl. Acad. Sci. USA *109*, 10498-10503.

Jo, S., Kim, T., Iyer, V.G., and Im, W. (2008). CHARMM-GUI: a web-based graphical user interface for CHARMM. J. Computat. Chem. *29*, 1859-1865.

Jo, S., Song, K.C., Desaire, H., MacKerell, A.D., Jr., and Im, W. (2011). Glycan Reader: automated sugar identification and simulation preparation for carbohydrates and glycoproteins. J. Computat. Chem. *32*, 3135-3141.

Kabsch, W. (1993). Automatic processing of rotation diffraction data from crystals of initially unknown symmetry and cell constants. J. Appl. Cryst. *26*, 795-800.

Kidmose, R.T., Laursen, N.S., Dobo, J., Kjaer, T.R., Sirotkina, S., Yatime, L., Sottrup-Jensen, L., Thiel, S., Gal, P., and Andersen, G.R. (2012). Structural basis for activation of the complement system by component C4 cleavage. Proc. Natl. Acad. Sci. USA *109*, 15425-15430.

Kjaer, T.R., Le le, T.M., Pedersen, J.S., Sander, B., Golas, M.M., Jensenius, J.C., Andersen, G.R., and Thiel, S. (2015). Structural insights into the initiating complex of the lectin pathway of complement activation. Structure *23*, 342-351.

Krissinel, E., and Henrick, K. (2007). Inference of macromolecular assemblies from crystalline state. J. Mol. Biol. *372*, 774-797.

Laue, T.M., Bhairavi, D.S., Ridgeway, T.M., and Pelletier, S.L. (1992). In Analytical Ultracentrifugation in Biochemistry and Polymers Science, S.E. Harding, A.J. Rowe, and J.C. Horton, eds. (Royal Society of Chemistry), pp. 90-125.

MacKerell, A.D., Bashford, D., Bellott, M., Dunbrack, R.L., Evanseck, J.D., Field, M.J., Fischer, S., Gao, J., Guo, H., Ha, S., *et al.* (1998). All-atom empirical potential for molecular modeling and dynamics studies of proteins. J. Phys. Chem. B *102*, 3586-3616.

Mackerell, A.D., Jr., Feig, M., and Brooks, C.L., 3rd (2004). Extending the treatment of backbone energetics in protein force fields: limitations of gas-phase quantum mechanics in reproducing protein conformational distributions in molecular dynamics simulations. J. Comput. Chem. *25*, 1400-1415.

McCoy, A.J., Grosse-Kunstleve, R.W., Adams, P.D., Winn, M.D., Storoni, L.C., and Read, R.J. (2007). Phaser crystallographic software. J. Appl. Cryst. *40*, 658-674.

Miller, A., Phillips, A., Gor, J., Wallis, R., and Perkins, S.J. (2012). Near-planar solution structures of mannose-binding lectin oligomers provide insight on the activation of the lectin pathway of complement. J. Biol. Chem. *287*, 3930-3945.

Nan, R., Gor, J., Lengyel, I., and Perkins, S.J. (2008). Uncontrolled zinc- and copper-induced oligomerisation of the human complement regulator factor H and its possible implications for function and disease. J. Mol. Biol. *384*, 1341-1352.

Okemefuna, A.I., Gilbert, H.E., Griggs, K.M., Ormsby, R.J., Gordon, D.L., and Perkins, S.J. (2008). The regulatory SCR-1/5 and cell surface-binding SCR-16/20 fragments of factor H reveal partially folded-back solution structures and different self-associative properties. J. Mol. Biol. *375*, 80-101.

Perkins, S.J. (1986). Protein volumes and hydration effects. The calculations of partial specific volumes, neutron scattering matchpoints and 280-nm absorption coefficients for proteins and glycoproteins from amino acid sequences. Eur. J. Biochem. *157*, 169-180.

Perkins, S.J. (2001). X-ray and neutron scattering analyses of hydration shells: a molecular interpretation based on sequence predictions and modelling fits. Biophys. Chem *93*, 129-139.

Perkins, S.J., Okemefuna, A.I., Nan, R., Li, K., and Bonner, A. (2009). Constrained solution scattering modelling of human antibodies and complement proteins reveals novel biological insights. J. Roy. Soc. Interface *6*, S679-696.

Perkins, S.J., Nan, R., Li, K., Khan, S., and Abe, Y. (2011). Analytical ultracentrifugation combined with X-ray and neutron scattering: Experiment and modelling. Methods *54*, 181-199.

Perkins, S.J., Wright, D.W., Zhang, H., Brookes, E.H., Chen, J., Irving, T.C., Krueger, S., Barlow, D.J., Edler, K.J., Scott, D.J. *et al.* (2016) Atomistic modelling of scattering data in the Collaborative Computational Project for Small Angle Scattering (CCP-SAS). J. App. Crystallogr. *In press.*

Pernot, P., Round, A., Barrett, R., De Maria Antolinos, A., Gobbo, A., Gordon, E., Huet, J., Kieffer, J., Lentini, M., Mattenet, M., *et al.* (2013). Upgraded ESRF BM29 beamline for SAXS on macromolecules in solution. J. Synch. Rad. *20*, 660-664.

Prosser, B.E., Johnson, S., Roversi, P., Herbert, A.P., Blaum, B.S., Tyrrell, J., Jowitt, T.A., Clark, S.J., Tarelli, E., Uhrin, D., *et al.* (2007). Structural basis for complement factor H linked age-related macular degeneration. J. Exp. Med. *204*, 2277-2283.

Raman, E.P., Guvench, O., and MacKerell, A.D., Jr. (2010). CHARMM additive all-atom force field for glycosidic linkages in carbohydrates involving furanoses. J. Phys. Chem. B *114*, 12981-12994.

Rayner, L.E., Hui, G.K., Gor, J., Heenan, R.K., Dalby, P.A., and Perkins, S.J. (2014). The Fab conformations in the solution structure of human immunoglobulin G4 (IgG4) restrict access to its Fc region: implications for functional activity. J. Biol. Chem. *289*, 20740-20756.

Rayner, L.E., Hui, G.K., Gor, J., Heenan, R.K., Dalby, P.A., and Perkins, S.J. (2015). The solution structures of two human IgG1 antibodies show conformational stability and accommodate their C1q and FcgammaR ligands. J. Biol. Chem. *290*, 8420-8438.

Rodriguez, E., Nan, R., Li, K., Gor, J., and Perkins, S.J. (2015). A revised mechanism for the activation of complement C3 to C3b: a molecular explanation of a disease-associated polymorphism. J. Biol. Chem. *290*, 2334-2350.

Round, A., Felisaz, F., Fodinger, L., Gobbo, A., Huet, J., Villard, C., Blanchet, C.E., Pernot, P., McSweeney, S., Roessle, M., *et al.* (2015). BioSAXS Sample Changer: a robotic sample changer for rapid and reliable high-throughput X-ray solution scattering experiments. Acta Cryst. D*71*, 67-75.

Roversi, P., Johnson, S., Caesar, J.J., McLean, F., Leath, K.J., Tsiftsoglou, S.A., Morgan, B.P., Harris, C.L., Sim, R.B., and Lea, S.M. (2011a). Structures of the rat complement regulator CrrY. Acta Cryst. F*67*, 739-743.

Roversi, P., Johnson, S., Caesar, J.J., McLean, F., Leath, K.J., Tsiftsoglou, S.A., Morgan, B.P., Harris, C.L., Sim, R.B., and Lea, S.M. (2011b). Structural basis for complement factor I control and its disease-associated sequence polymorphisms. Proc. Natl. Acad. Sci. USA *108*, 12839-12844.

Sali, A., and Blundell, T.L. (1993). Comparative protein modelling by satisfaction of spatial restraints. J. Mol. Biol. *234*, 779-815.

Saphire, E.O., Parren, P.W., Pantophlet, R., Zwick, M.B., Morris, G.M., Rudd, P.M., Dwek, R.A., Stanfield, R.L., Burton, D.R., and Wilson, I.A. (2001). Crystal structure of a neutralizing human IGG against HIV-1: a template for vaccine design. Science *293*, 1155-1159.

Schuck, P. (1998). Sedimentation analysis of noninteracting and self-associating solutes using numerical solutions to the Lamm equation. Biophys. J. *75*, 1503-1512.

Schuck, P. (2000). Size-distribution analysis of macromolecules by sedimentation velocity ultracentrifugation and lamm equation modeling. Biophys. J. *78*, 1606-1619.

Semenyuk, A.V., and Svergun, D.I. (1991). GNOM – a program package for small-angle scattering data-processing. . J. Appl. Crystallogr. *48*, 953-961.

Skjoedt, M.O., Roversi, P., Hummelshoj, T., Palarasah, Y., Rosbjerg, A., Johnson, S., Lea, S.M., and Garred, P. (2012). Crystal structure and functional characterization of the complement regulator mannose-binding lectin (MBL)/ficolin-associated protein-1 (MAP-1). J. Biol. Chem. *287*, 32913-32921.

Teillet, F., Gaboriaud, C., Lacroix, M., Martin, L., Arlaud, G.J., and Thielens, N.M. (2008). Crystal structure of the CUB1-EGF-CUB2 domain of human MASP-1/3 and identification of its interaction sites with mannan-binding lectin and ficolins. J. Biol. Chem. *283*, 25715-25724.

Wallis, R., and Dodd, R.B. (2000). Interaction of mannose-binding protein with associated serine proteases: effects of naturally occurring mutations. J. Biol. Chem. *275*, 30962-30969.

Wright, D.W., and Perkins, S.J. (2015). SCT: a suite of programs for comparing atomistic models with small-angle scattering data. J. Appl. Cryst. *48*, 953-961.

Wu, J., Wu, Y.Q., Ricklin, D., Janssen, B.J., Lambris, J.D., and Gros, P. (2009). Structure of complement fragment C3b-factor H and implications for host protection by complement regulators. Nat. Immunol. *10*, 728-733.
